# Supplementary material for: Effect of accelerated postoperative rehabilitation after tibial tubercle distalisation: A randomised controlled trial protocol
Source: PLoS One. 2024 Jul 11;19(7):e0304075. doi: 10.1371/journal.pone.0304075 (PMC11239065; doi:10.1371/journal.pone.0304075)
Supplement: S1 File — Accelerated Rehabilitation Program. (PDF) [file pone.0304075.s001.pdf]

S1 File. Protocol 1. Accelerated rehabilitation program.

Accelerated rehabilitation

	Week														
Program no	0	1	2	3	4	5	6	7	8	9	10	11	12	13	14
PT_1	x	x													
PT_2			x	x	x	x									
PT_3							x	x							
PT_4									x	x					
PT_5											x	x	x	x	
PT_6															x

See Personal Exercise Program 1-6 (PT_1...PT_6) from supplementary material.
