# Supplementary material for: Effect of accelerated postoperative rehabilitation after tibial tubercle distalisation: A randomised controlled trial protocol
Source: PLoS One. 2024 Jul 11;19(7):e0304075. doi: 10.1371/journal.pone.0304075 (PMC11239065; doi:10.1371/journal.pone.0304075)
Supplement: S3 File — Personal Exercise Program 1. (PDF) [file pone.0304075.s003.pdf]

Personal exercise program

Program1*

Pihlajalinna Oy
Pihlajalinna Kelloportti
Kelloportinkatu 1, 33100, Tampere, Finland

Laatija	Erkki Nilkku
Harjoittelu alkaa	27.5.2024

During the first days (2-3 days) move only moderately, so that the swelling does not build up too much. To treat swelling and pain use an elevated position and cold treatment. Cold treatment varies 2-3 hours for 15-20 min at a time, especially during the first one or two days. Also remember to pump the ankle 20 reps at the time. Do exercises 2 times per day.

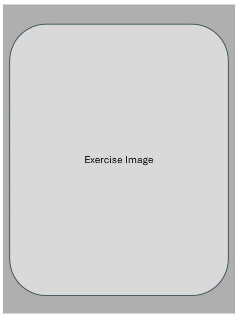

Straight Leg Raise in Supine

Lie on your back with one leg bent and foot on the floor, the other leg is straight.

Bend the ankle of the straight leg, contract the muscles of your front thigh and lift the leg off the floor keeping it straight.
In a controlled manner, return to the starting position.

Repeat 12 times.

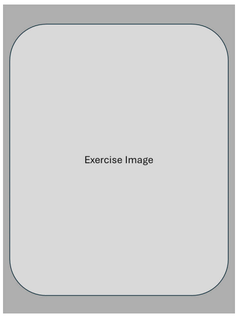

Knee End-range Extension in Supine

Lie on your back with one leg bent and the other leg straight. Place a towel roll under the straight knee.

Bend your ankle and straighten the knee using your front thigh muscles.
Keep the back of your knee against the towel roll.
Keep the tension for a moment and then relax.

Repeat 12 times.

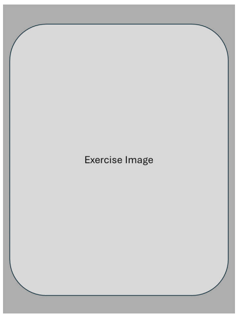

Isometric Knee Extension in Supine with Both Legs

Lie on your back with your knees straight.

Bend your ankles and press the back of your knees against the floor by using your front thigh muscles. At the same time squeeze your buttock muscles.
Hold the tension for a moment and then relax.

Repeat 12 times.

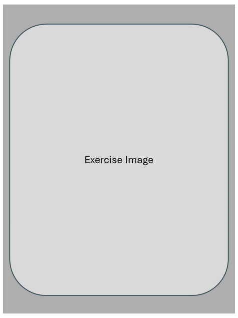

Seated Knee End-Range Extension Stretch with Ice

Place a rolled towel under your ankle.

Place an ice pack on your knee. Let the knee straighten.

Hold for 5 min

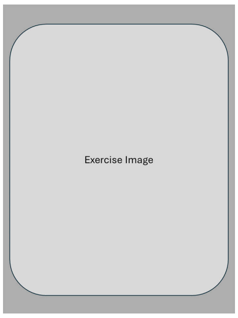

Seated Heel Slides

Sit with a towel under one foot.

Bend and straighten your knee by sliding your foot along the floor.

Repeat 12 times.

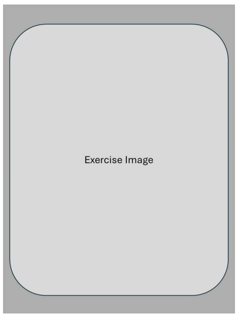

Modified Clam with Hips in 90 Degrees

Start by lying on your side with your hips and knees bent to 90 degrees. Take support with your hand from the floor in front of you. Press your hand against the floor and lift your lower side slightly off the floor.

Keep your feet together and turn from your hip to lift the knee upwards. Keep your pelvis still. In a controlled manner lower the knee back down. Feel the muscles in the side of your buttocks working.

Repeat 12 times.
