# Supplementary material for: Effect of accelerated postoperative rehabilitation after tibial tubercle distalisation: A randomised controlled trial protocol
Source: PLoS One. 2024 Jul 11;19(7):e0304075. doi: 10.1371/journal.pone.0304075 (PMC11239065; doi:10.1371/journal.pone.0304075)
Supplement: S4 File — Personal Exercise Program 2. (PDF) [file pone.0304075.s004.pdf]

Personal exercise program

personal exercise program 2

Pihlajalinna Oy

Pihlajalinna Kelloportti
Kelloportinkatu 1, 33100, Tampere, Finland

Laatija Erkki Nilkku
Harjoittelu alkaa 21.5.2024

This training period starts after the first orthopedic control. Do the exercises daily. During exercising you should not feel strong pain that exceeds NRS 0-10 as sensation 4. If the exercises cause more pain than 4 contact the attending physiotherapist.

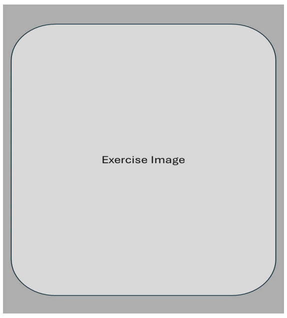

Hip Abduction

Lie on your side with the lower leg bent and upper leg straight. Support your head with your arm.

Lift the leg upwards using your buttocks and then lower it back to the starting position. Keep the leg in line with your body.

Repeat 7 times. Do 3 sets

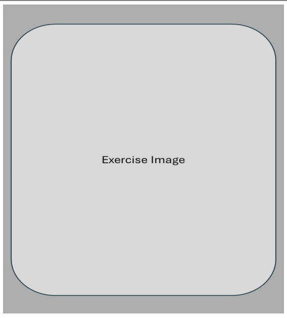

Single-leg Standing

Balance on one leg.

Remember to stand tall, with weight evenly on your foot and toes pointing forwards.

Repeat 7 times. Do 3 sets

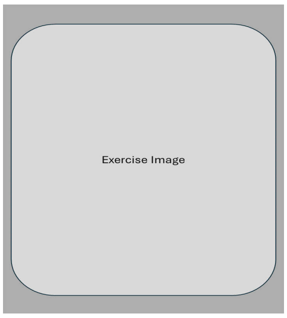

Seated Heel Slides

Sit with a towel under one foot.

Bend and straighten your knee by sliding your foot along the floor.

Repeat 7 times. Do 3 sets

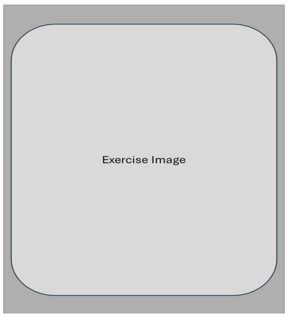

Seated Knee End-Range Extension Stretch

Place a rolled towel under your ankle.

Straighten your knee by tightening your thigh muscles. Try to touch the floor with the back of your knee.

Hold for 10s
Repeat 7 times. Do 3 sets

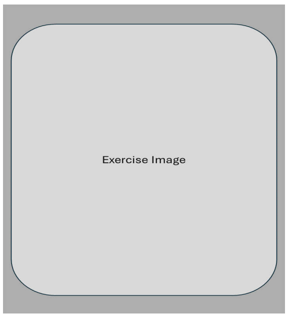

Active Knee End-range Extension

Start by lying on your stomach on the floor with your legs straight. Bend one ankle and support the toes against the floor.

Keep toes on the floor and straighten the knee so that it lifts off the floor and hold. Then relax and lower the knee back onto the floor.

Repeat 7 times.Do 3 sets

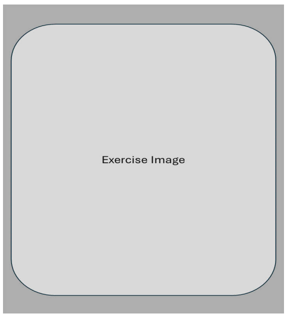

Hip Extension

Lie on your stomach.

Stretch your leg as long as possible and lift it off the floor. Lower the leg back on the floor. Repeat with the other side.

Repeat 7 times. Do 3 sets

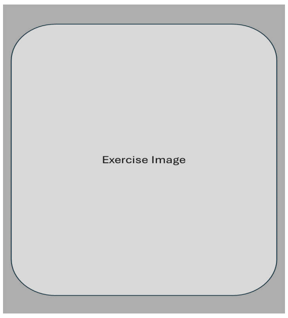

Balanced Standing - Balanced Weight Bearing

Stand with your legs apart and feet parallel.

Transfer your weight from one leg to the other. Remember to maintain full controlled knee extension.

Repeat 7 times. Do 3 sets

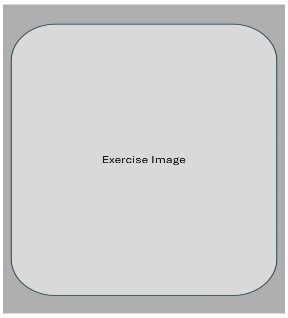

Resisted Knee End-range Extension in Standing

Stand up right with your feet approximately hip-width apart and weight distributed evenly between your feet. The loop of an exercise band in placed around the back of the knee you are training. The other end of the band is securely attached in front of you.

Let the heel rise off the floor and allow the pull from the band to bend your knee.
Straighten your knee by pressing your heel back to the floor and contracting your front thigh muscles.

Repeat 7 times.

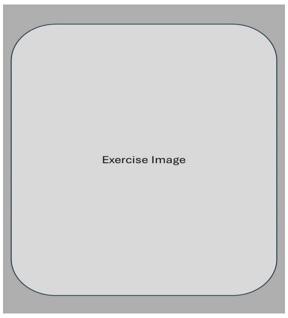

Heel Raise with Quad Activation

Start by standing with the balls of your feet on the edge of a step (or a thick book). An exercise band is placed behind one knee and atached securely in front with the slack taken off.

Rise onto your toes. Do not bend the knee. Feel the front thigh muscles working. Lower your heels back onto the floor.

Repeat 7 times.
