# Supplementary material for: Effect of accelerated postoperative rehabilitation after tibial tubercle distalisation: A randomised controlled trial protocol
Source: PLoS One. 2024 Jul 11;19(7):e0304075. doi: 10.1371/journal.pone.0304075 (PMC11239065; doi:10.1371/journal.pone.0304075)
Supplement: S5 File — Personal Exercise Program 3. (PDF) [file pone.0304075.s005.pdf]

Personal exercise program

personal exercise program 3

Pihlajalinna Oy
Pihlajalinna Kelloportti
Kelloportinkatu 1, 33100, Tampere, Finland

Laatija	Erkki Nilkku
Harjoittelu alkaa	21.5.2024

This training period starts after the first orthopedic control. Do the exercises daily. During exercising you should not feel strong pain that exceeds NRS 0-10 as sensation 4. If the exercises cause more pain than 4 contact the attending physiotherapist.

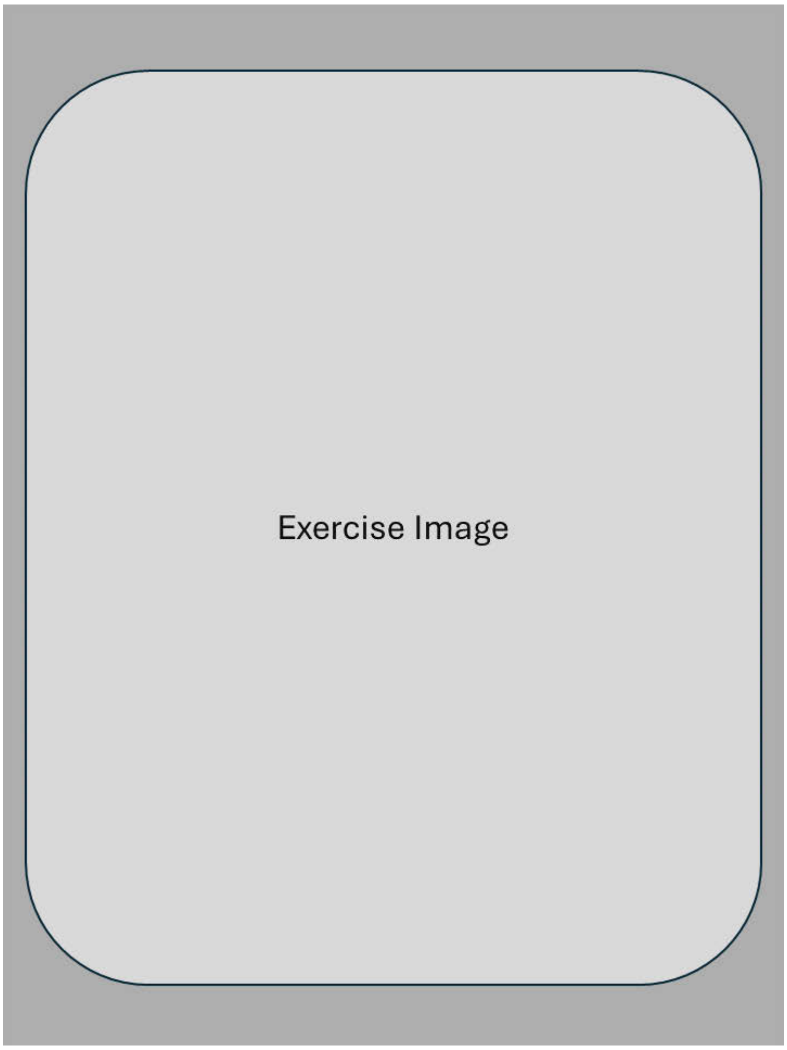

Stationary Cycling

Sit up straight on a stationary bike that has the seat adjusted to your height.

Start pedalling and select desired exercise option from the menu or just add resistance. Keep your neck and shoulder region relaxed.

Continue for 10min .

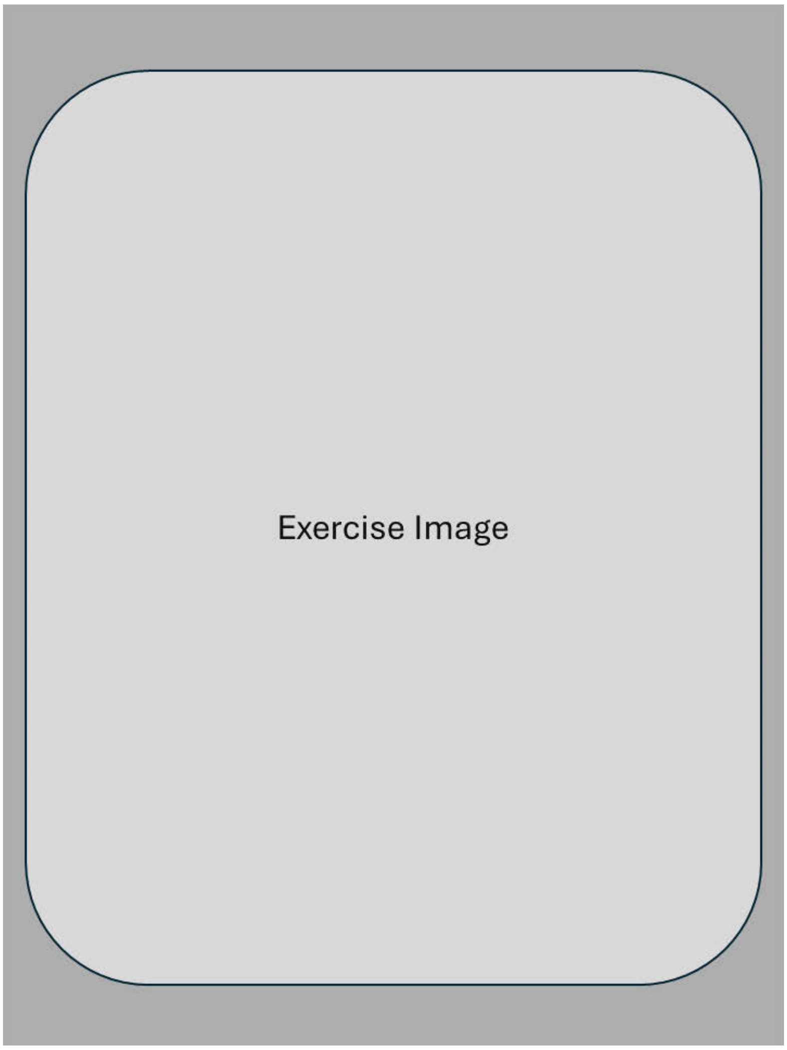

Squat

Stand tall with feet slightly wider than hip-width apart. Toes pointing forward or turned a few degrees outwards. Keep your chest up and your spine and neck in a neutral position.

Squat down by sitting back and bring your arms forward. Push back up through the heels, chest up, and straighten your hips.

Note:
- Keep your hips, knees and toes aligned and don't let your lower back round.
- Keep your weight evenly on your whole foot.

Repeat ___7___ times. Do 3 sets

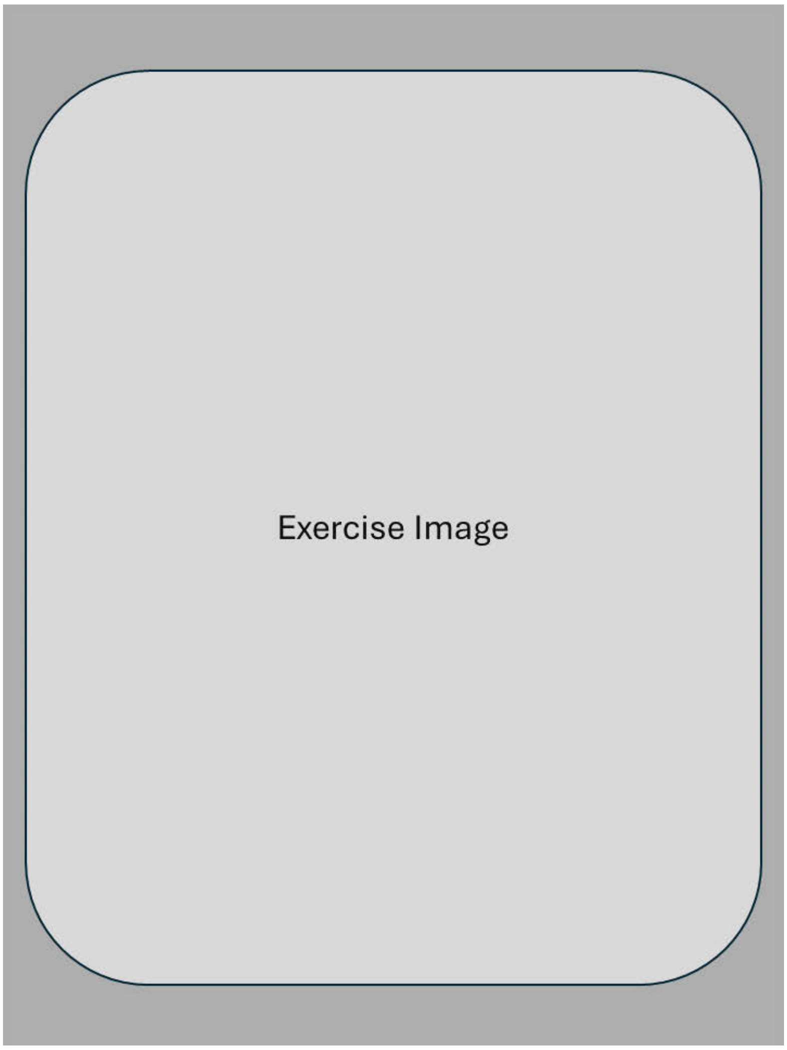

Lateral Step Up

Start by standing sideways next to a step, with one leg on the step.

Step up and straighten your hip and knee. Maintain hip-knee-toes alignment. Return to the starting position.

Repeat 7 times. Do 3 sets

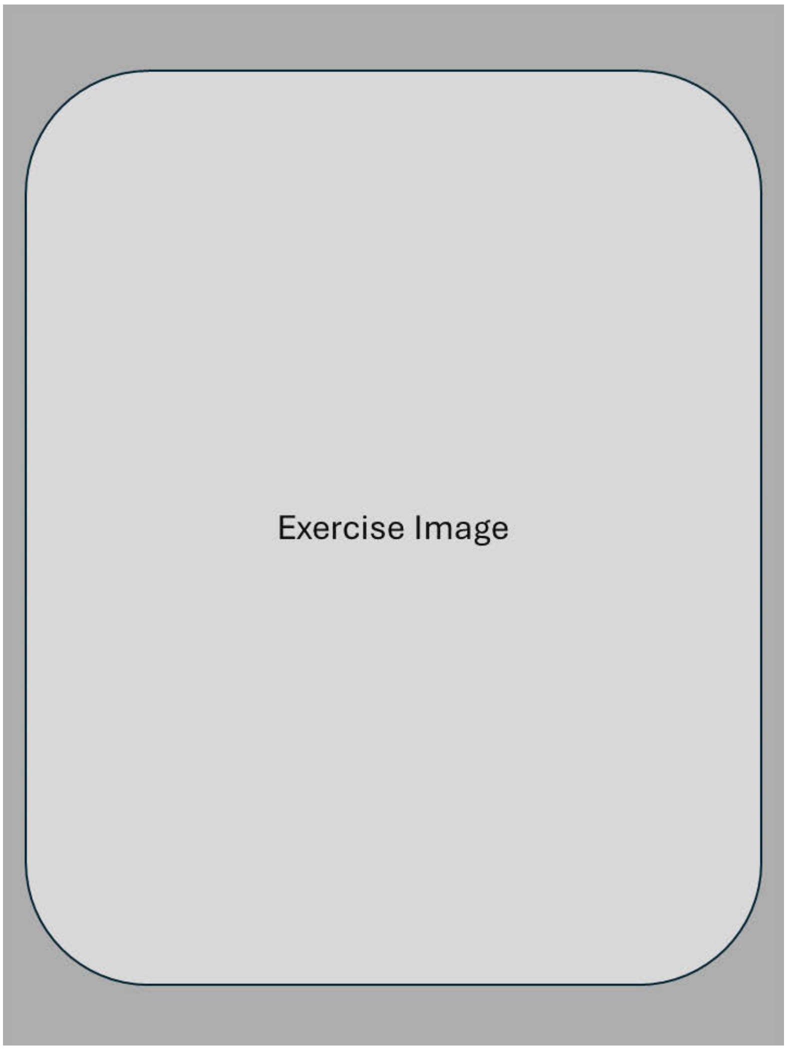

Static Wall Squat

Stand tall and lean your back against a wall and move your feet forwards, so that when you squat down your knees stay over or behind your ankles.

Squat down and hold the position for a moment. Push back up to starting position.

Hold for 40s. Repeat 5 times

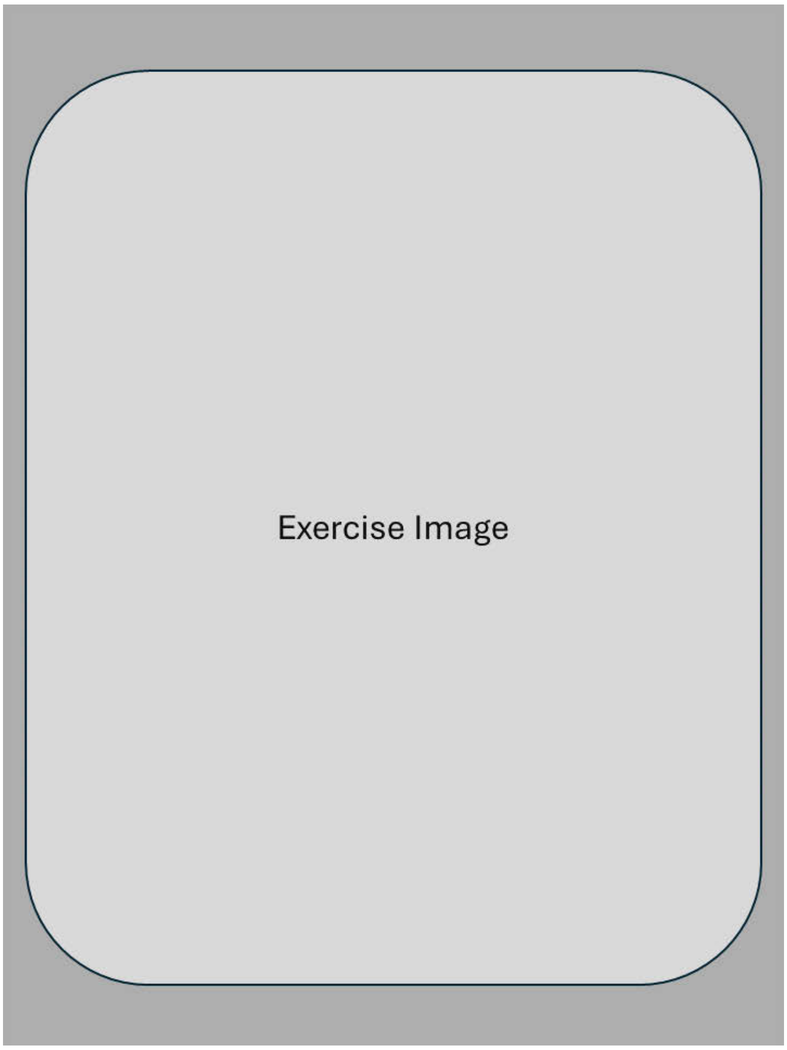

Bridge

Lie on your back, with knees bent and feet hip-width apart.

Draw in your abdominals and tighten your buttocks. Tilt your pelvis backwards and lift your pelvis and back up one vertebrae at a time. Lift only as high as you can while maintaining the pelvis position. Lower your pelvis down in a controlled manner.

Note: Don't let your lower back arch during the lift.

Repeat 7 times. Do 3 sets

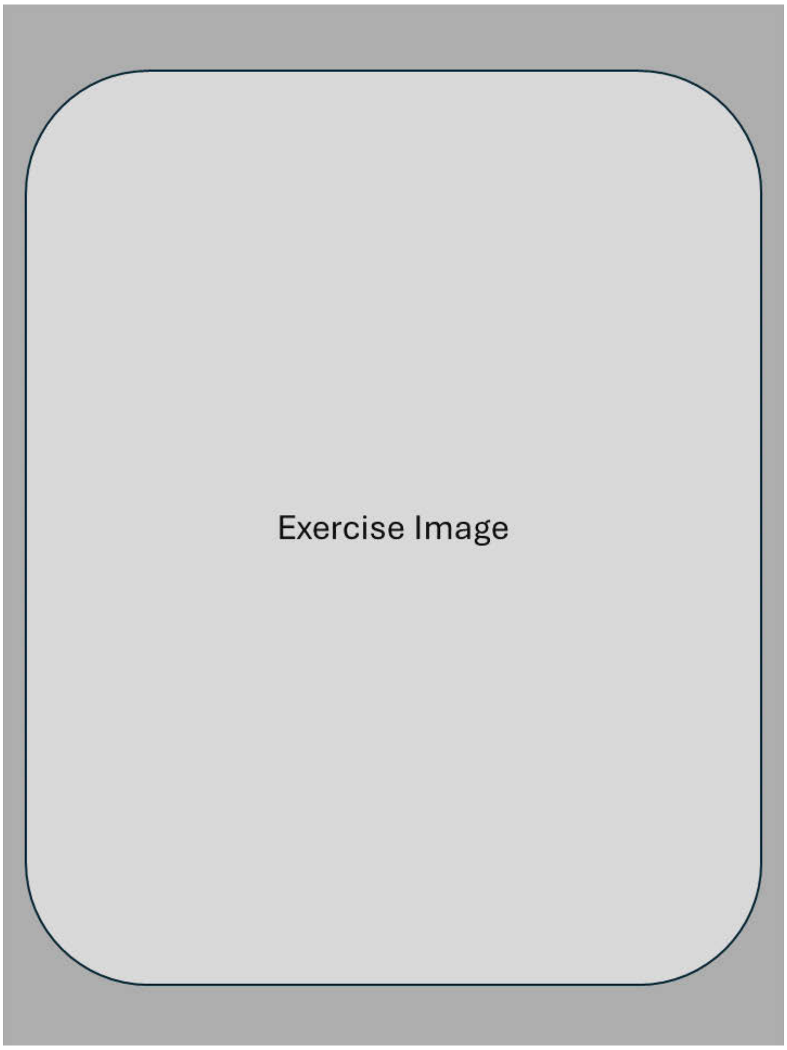

Hip Abduction

Lie on your side with the lower leg bent and upper leg straight. Support your head with your am.

Lift the leg upwards using your buttocks and then lower it back to the starting position. Keep the leg in line with your body.

Repeat ___7___ times. Do 3 sets

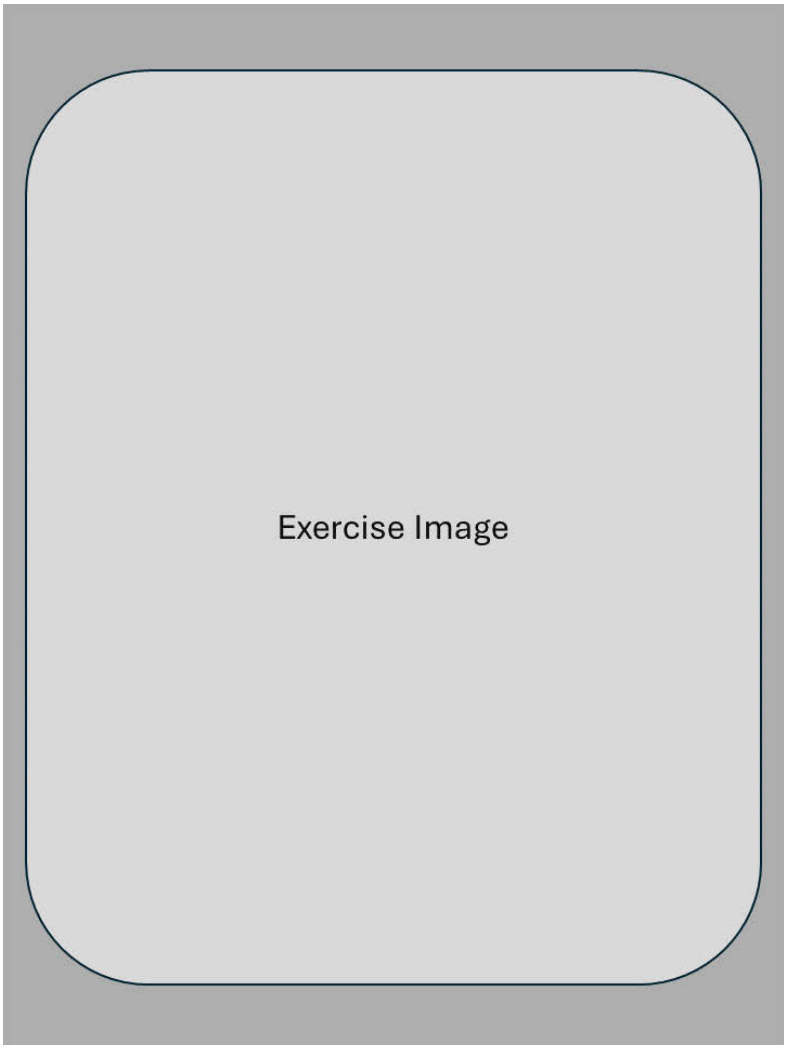

Hamstring Stretch with Exercise Band

Sit on the floor and put an exercise band around one foot and secure the ends in your hands. Lie on your back, bend one leg on the floor and bring the other foot towards the ceiling with your knee slightly bent.

Straighten the knee against the resistance from the exercise band. Then relax and let the knee bend again.

Hold for ___10___ seconds. Repeat 10 times

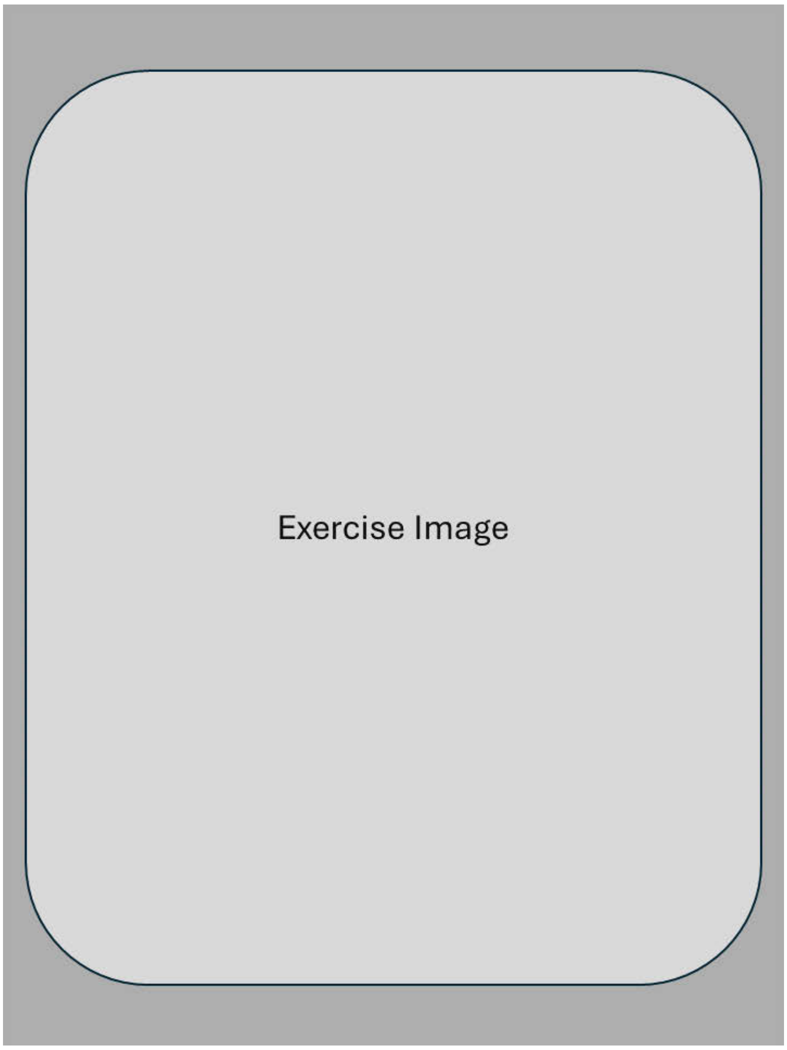

Assisted Knee Flexion

Sit on the floor. Your back can be supported. Place a band/belt around your foot and firmly hold both ends.

Bend your knee as far as you can and gently assist the bend with the band. Bend the knee until you feel a stretch in your knee area and hold. Lower the leg back to the starting position in a controlled manner.

Hold for 10 seconds.
Repeat 10 times.

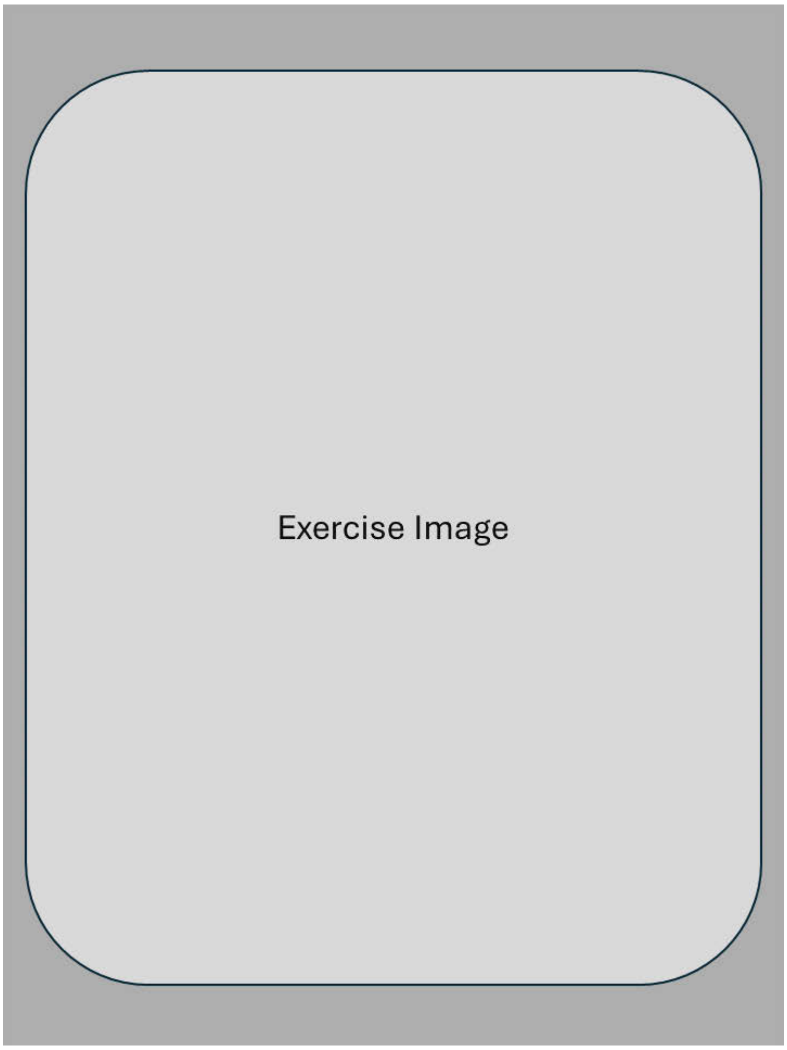

Lunges to Different Directions

Start by standing.

In turns, with one leg, take a step
- forwards and towards one side,
- straight forwards and
- forwards and cross the midline.

Remember to maintain hip-knee-toes alignment.

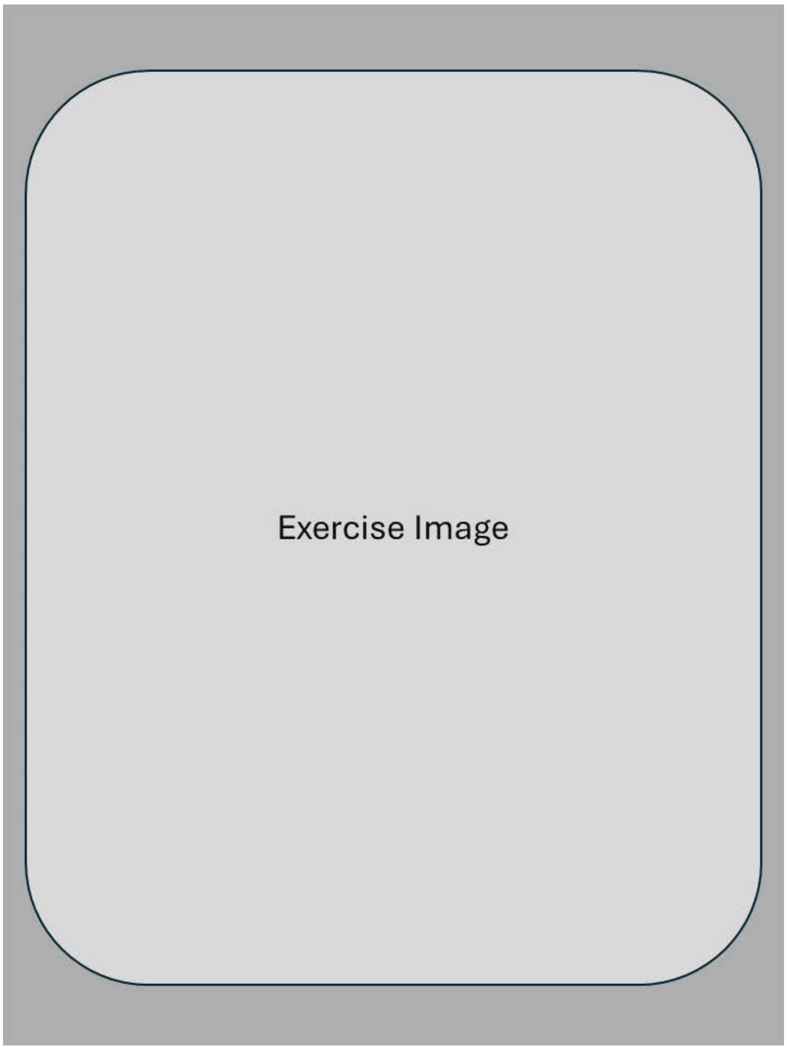

Take a step straight forwards...Repeat 7 times
. Do 3 sets

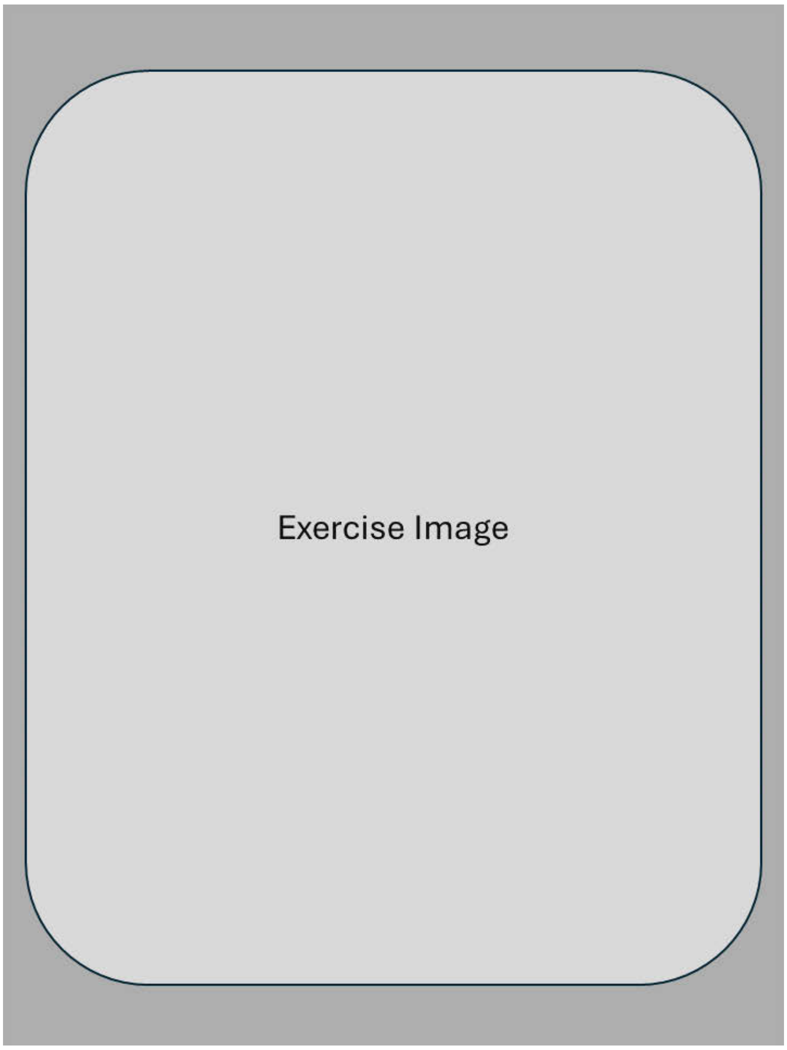

Take a step forwards and cross the midline.

Repeat 7 times. Do 3 sets
