# Supplementary material for: Effect of accelerated postoperative rehabilitation after tibial tubercle distalisation: A randomised controlled trial protocol
Source: PLoS One. 2024 Jul 11;19(7):e0304075. doi: 10.1371/journal.pone.0304075 (PMC11239065; doi:10.1371/journal.pone.0304075)
Supplement: S6 File — Personal Exercise Program 4. (PDF) [file pone.0304075.s006.pdf]

Personal exercise program

Personal exercise program 4

Pihlajalinna Oy
Pihlajalinna Kelloportti
Kelloportinkatu 1, 33100, Tampere, Finland

Laatija	Erkki Nilkku
Harjoittelu alkaa	21.5.2024

Do the exercises every other day. Try to do exercises as carefully as possible. If you feel severe pain in your knee during exercise, contact your attending physiotherapist.

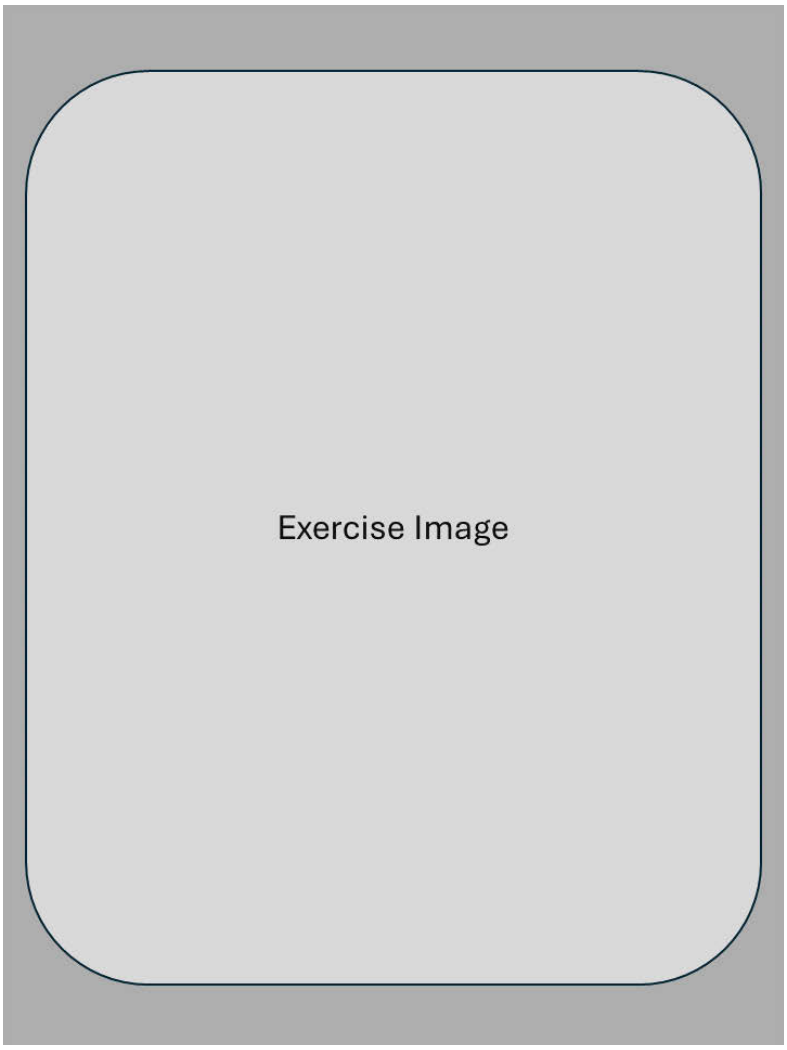

Stationary Cycling

Sit up straight on a stationary bike that has the seat adjusted to your height.

Start pedalling and select desired exercise option from the menu or just add resistance. Keep your neck and shoulder region relaxed.

Continue for 10 min .

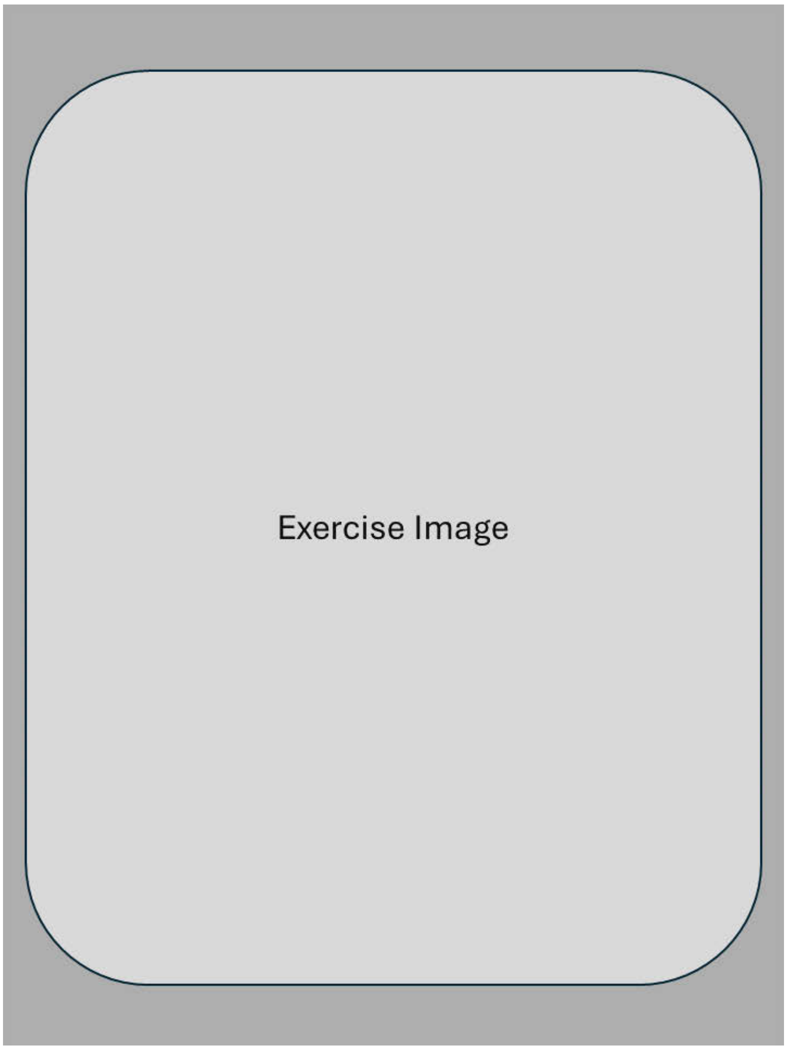

Single-leg Squat with Hip Abduction (Side Lunge)

Start by standing with an exercise band loop around both feet.

Squat down with one leg and at the same time slide the other leg sideways keeping the knee straight and toes on the floor. Maintain hip-knee-foot alignment. Return to the starting position.

Repeat ___7___ times. Do 3 sets

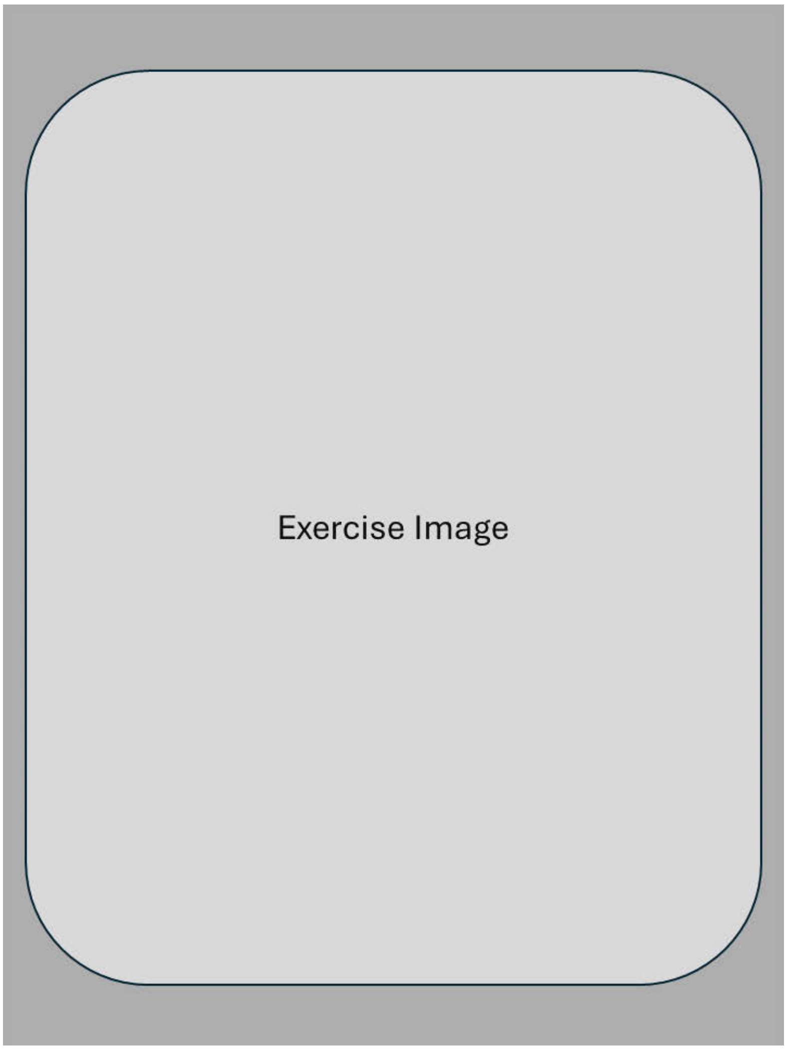

Glute Walk

Stand tall, with an exercise or loop band around your legs (slightly above or under the knee joint).

Take a hip-width stance so that the band tightens and squat down. In this position, step to the side then return, keeping the band tight at all times.

Repeat ___7___ times. do 3 sets

Note:
Focus on using your buttock muscles.
You can also step forwards and backwards, but remember to keep feet at least hip width apart.

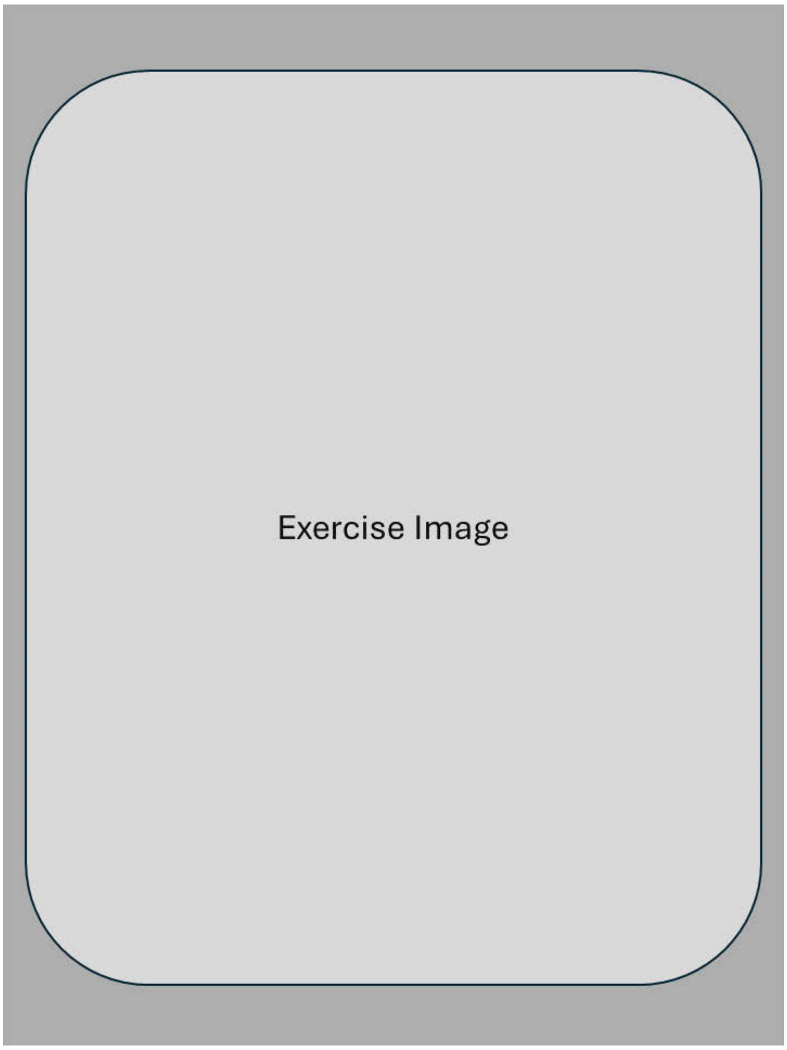

Squat with Mini Band around Legs

Start by standing with a mini band around your knees (slightly above or under). Your legs are approximately hip-width apart or slightly wider and toes pointing forwards or slightly outwards. Arms can be crossed over chest or placed on your hips or they can move along with the squat.

Squat down; move your hips back and bend your legs. Press your knees outwards against the resistance from the band to keep toes, ankles, knees and hips aligned. Feel the tension in your thighs and buttocks. Push back up to standing and straighten your knees and hips.

Repeat ___7___ times. Do 3 sets

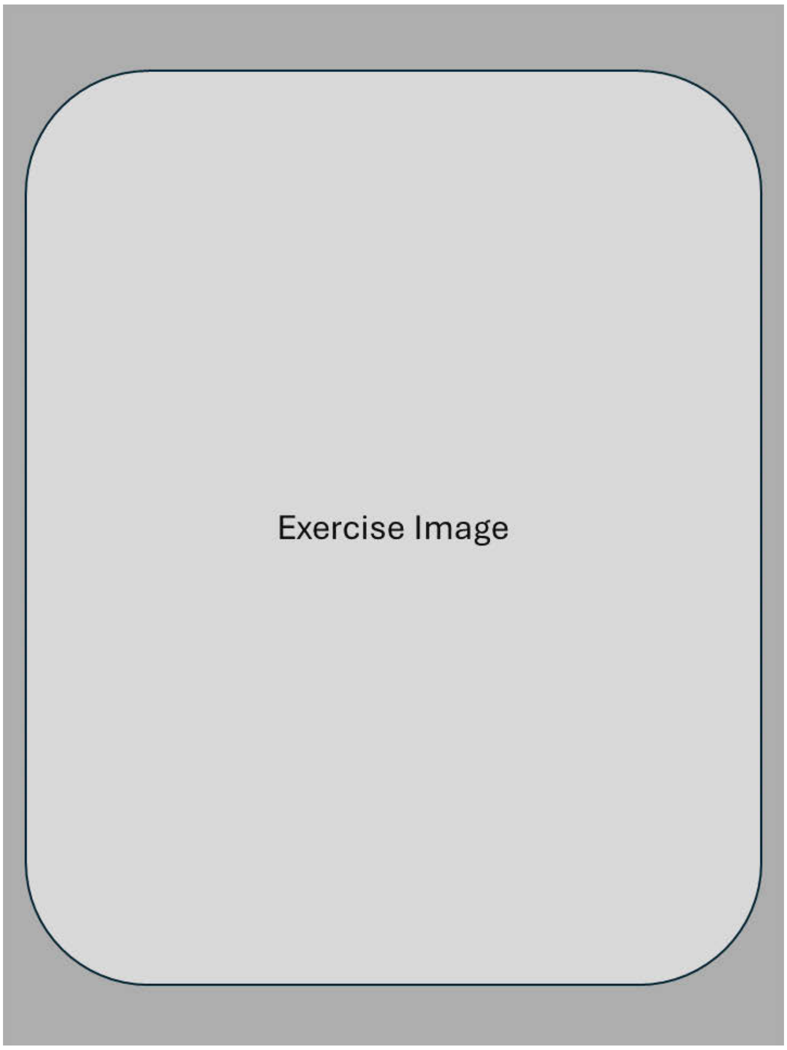

Step Down

Stand on a step and bring the other leg backwards.

Squat down to touch the floor with the rear leg. Squat only as deep as you can control the alignment of the supportive leg. The hip, knee and ankle should stay in a straight line and pelvis horizontal.

Repeat 7 times. Do 3 sets

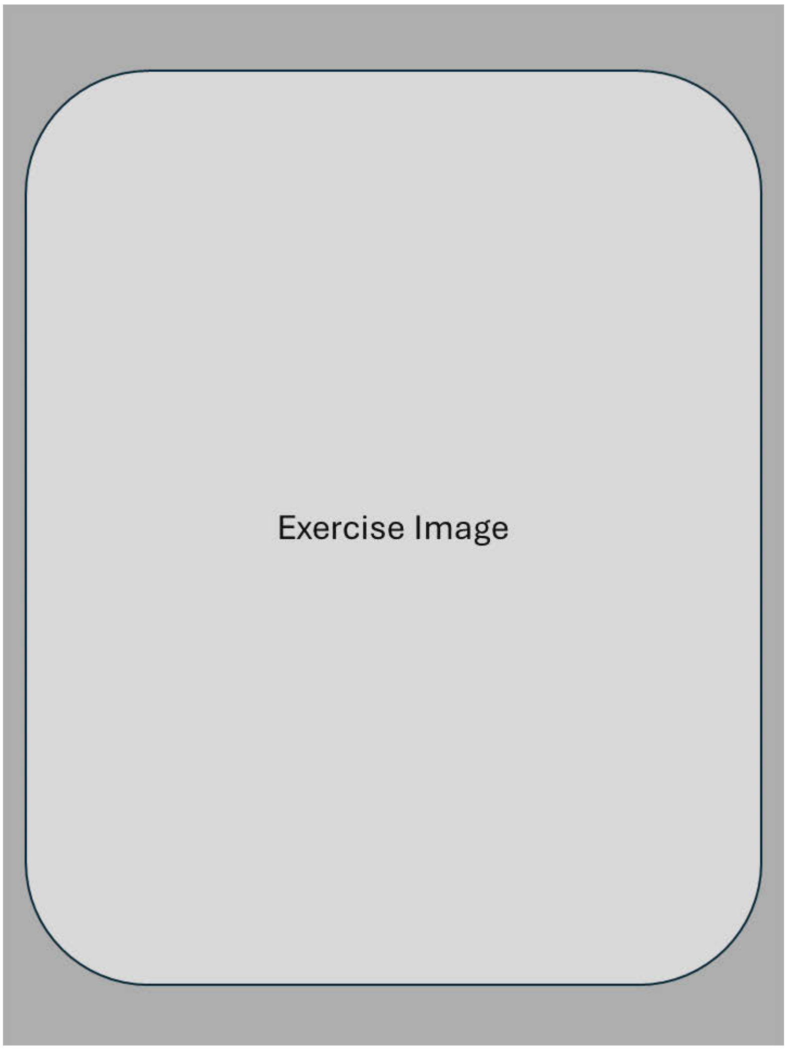

Lateral Step Down

Stand sideways on the edge of a step so that your outer leg hangs off the edge.

Squat down to touch the floor with the outer leg. Squat only as deep as you can control the alignment of the supportive leg. The hip, knee and ankle should stay in a straight line and pelvis horizontal.

Repeat 7 times. Do 3 sets

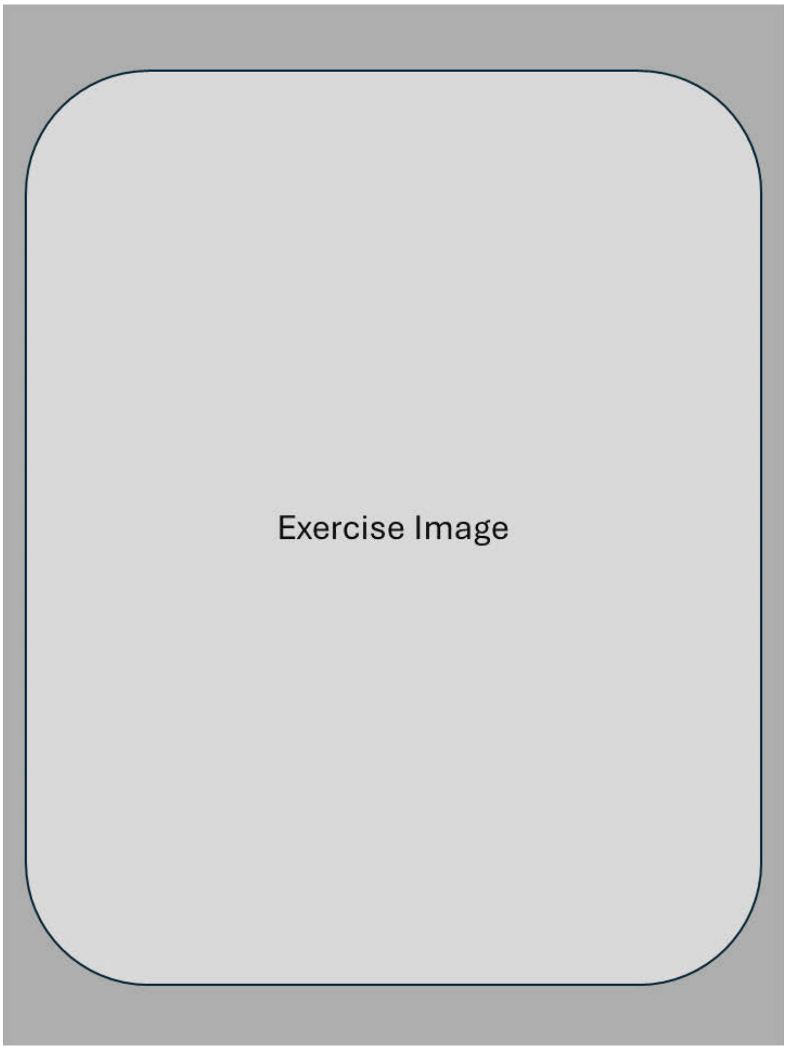

Step Up Knee Drives with Quad Activation

Start by standing with one leg on a step and an exercise band looped behind the knee and secured to the front (pulling the knee forwards).

Step up using the front leg and drive the knee of the other leg forwards. Ensure full hip and knee extension on the stance leg. Return to starting position.

Repeat 7 times. Do 3 sets

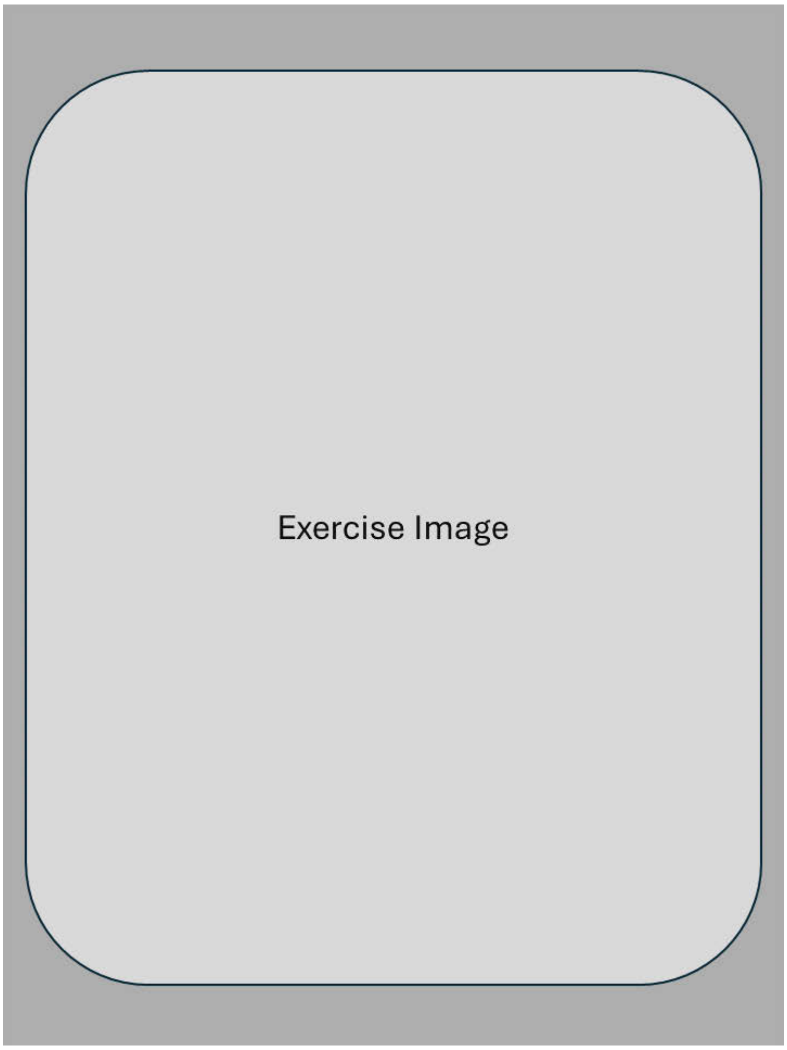

Lateral Step Down with an Exercise Band

Stand on the edge of a step with an exercise band around your leg (just under or above knee joint) and attached firmly to the side (band pulls your knee inwards).

Squat down and let your free leg brush the floor. Maintain hip, knee and 2nd toe alignment in the leg on the step by squeezing your buttocks.

Repeat 7 times. Do 3 sets

Note:
Don't let the band pull your knee inwards.
Don't let your pelvis tilt.

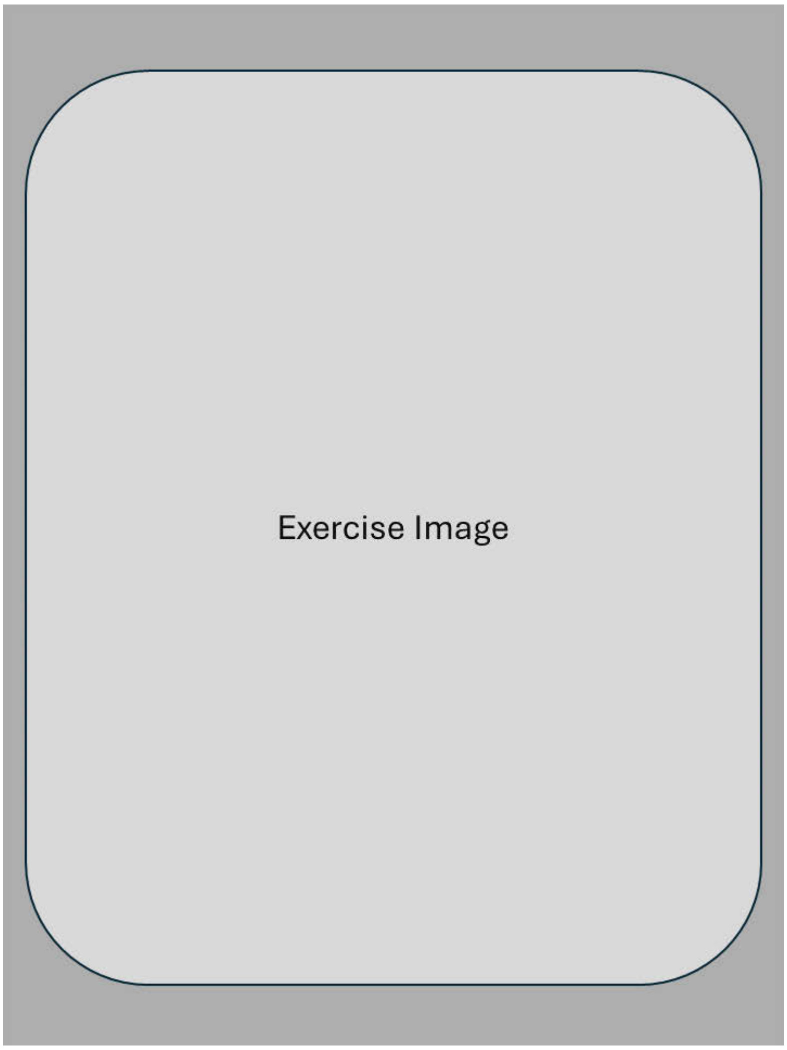

Single-leg Bridge

Lie on your back with one leg bent and foot placed on a step. The other leg is bent and held in the air.

Flatten your lower back against the floor. Squeeze your buttocks, lift your pelvis and straighten your hip. Return to the starting position.

Repeat 7 times. Do 3 sets
