# Supplementary material for: Effect of accelerated postoperative rehabilitation after tibial tubercle distalisation: A randomised controlled trial protocol
Source: PLoS One. 2024 Jul 11;19(7):e0304075. doi: 10.1371/journal.pone.0304075 (PMC11239065; doi:10.1371/journal.pone.0304075)
Supplement: S7 File — Personal Exercise Program 5. (PDF) [file pone.0304075.s007.pdf]

Personal exercise program

Personal exercise program5*

Pihlajalinna Oy

Pihlajalinna Kelloportti
Kelloportinkatu 1, 33100, Tampere, Finland

Laatija	Erkki Nilkku
Harjoittelu alkaa	21.5.2024

Do exercises 3 times a week

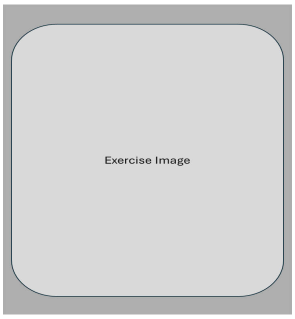

Stationary Cycling

Sit up straight on a stationary bike that has the seat adjusted to your height.

Start pedalling and select desired exercise option from the menu or just add resistance. Keep your neck and shoulder region relaxed.

Continue for 15 min .

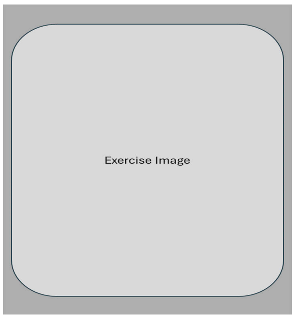

Overhead with the plate

Stand tall, with your feet slightly wider than hip-width apart. Hold a weight plate with both hands, arms hanging straight and in front of the body.

Squat down. Keep your knees aligned with your toes and weight evenly on both feet. Slightly lean forward from hips. Lower the weight straight down between your feet. Then push back up to standing and continue up to your toes. Bring the weight up to straight arms straight over your head. Keep the weight close to your body during the lift. Return back to the starting position.Repeat 12 times. Do 3 sets

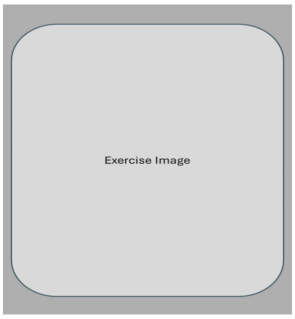

One Arm High Row with a Cable

Stand tall holding a single handle that is attached to a cable with your arm straight. Slightly tilt your trunk forwards and lift and straighten the same side leg backwards.

Pull the cable by bringing your elbow high and backwards. At the same time, bring the same side knee forwards.
In a controlled manner let the arm and leg straighten back to the starting position. Maintain your balance. Repeat 12 times. Do 3 sets

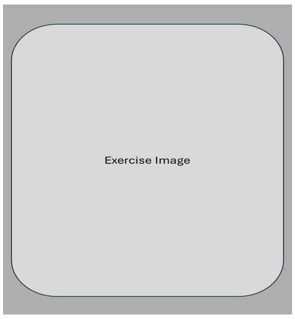

Diagonal One Arm Press with a Cable

Stand tall, with your back towards a cable machine, holding a single handle that is attached to a high cable. Your arm is bent, and the same side knee is lifted high.

Let your trunk tilt forwards while you press the arm holding the handle forwards and down. At the same time straighten the bent leg backwards. In a controlled manner return to the starting position.Repeat 12 times. Do 3 sets

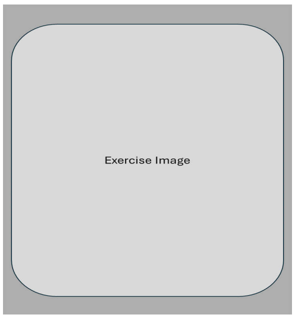

Single-leg Deadlift

Stand tall holding a weight in one hand and leg of the same side lifted up.

Keep the knee of the standing leg slightly bent and bend forward from your hip. At the same time straighten your other leg backwards and lower the weight towards the floor.

Use your hamstrings and buttock muscles to return to single-leg standing.

Note: Keep trunk active and maintain the neutral position of your spine throughout the exercise.

Repeat 12 times. Do 3 sets

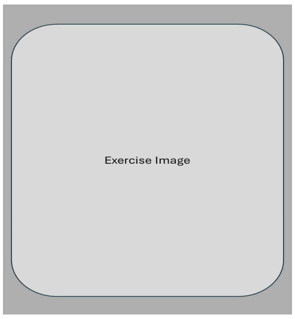

Squat with Dumbbells

Stand tall with your feet slightly wider apart than your hips, holding dumbbells in both hands.

Squat down by sitting back and keep your trunk in an upright position. Push back up and straighten your hips.

Note:
- Keep hips, knees and toes aligned.
- Keep your weight evenly distributed between your forefoot and heel.

Repeat 12 times. Do 3 sets

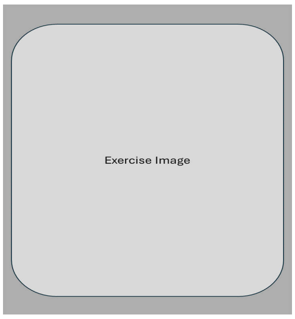

Seated Leg Curl

Sit up straight on a leg curl machine with your knees straight and the resistance lever behind your ankles.

Bend your knees and bring heels towards your buttocks. In a controlled manner let your knees straighten back to the starting position.

Note: Some machines also have another support that should be on the front side, just under your knees.

Repeat 12 times. Do 3 sets

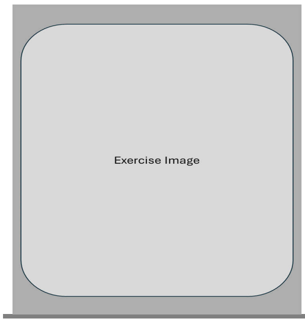

Knee Extension in a Machine

Sit up straight on a knee extension machine with your knees bent and the resistance lever in front of your ankles.

Straighten your knees. In a controlled manner let your knees bend back to the starting position. Do the knee movement 0-30 grade angle.

Repeat ___12___ times. Do 3 sets

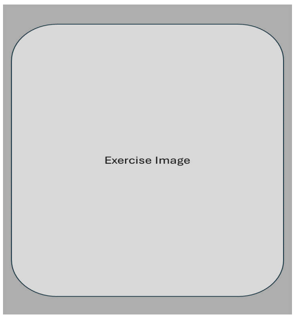

Leg Press

Sit up straight on a leg press machine, with your feet placed hip-width apart on the platform. Release the safety locks and lower down to a squat and lock the position.

Push through your heels and straighten your knees. Actively push your knees out, to avoid them collapsing in. In a controlled manner lower yourself back to the squat.

Note: The weight is evenly distributed between the heels and forefeet.

Repeat ___12___ times. Do 3 sets

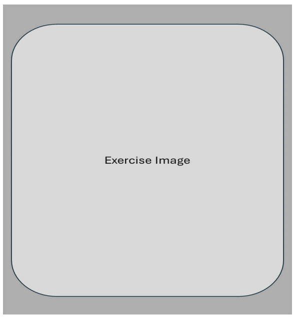

Calf Press

Sit up straight on a leg press machine with the balls of your feet placed near the lower edge of the leg press platform with the heels hanging off.

With your knees straight, press against the platform and straighten your ankles. In a controlled manner let your ankles bend, while keeping your knees straight.

Repeat ___12___ times. Do 3 sets
