# Supplementary material for: Effect of accelerated postoperative rehabilitation after tibial tubercle distalisation: A randomised controlled trial protocol
Source: PLoS One. 2024 Jul 11;19(7):e0304075. doi: 10.1371/journal.pone.0304075 (PMC11239065; doi:10.1371/journal.pone.0304075)
Supplement: S8 File — Personal Exercise Program 6. (PDF) [file pone.0304075.s008.pdf]

Personal exercise program

Personal exercise program 6*

Pihlajalinna Oy
Pihlajalinna Kelloportti
Kelloportinkatu 1, 33100, Tampere, Finland

Laatija	Erkki Nilkku
Harjoittelu alkaa	21.5.2024

Do exercises 2 times a week

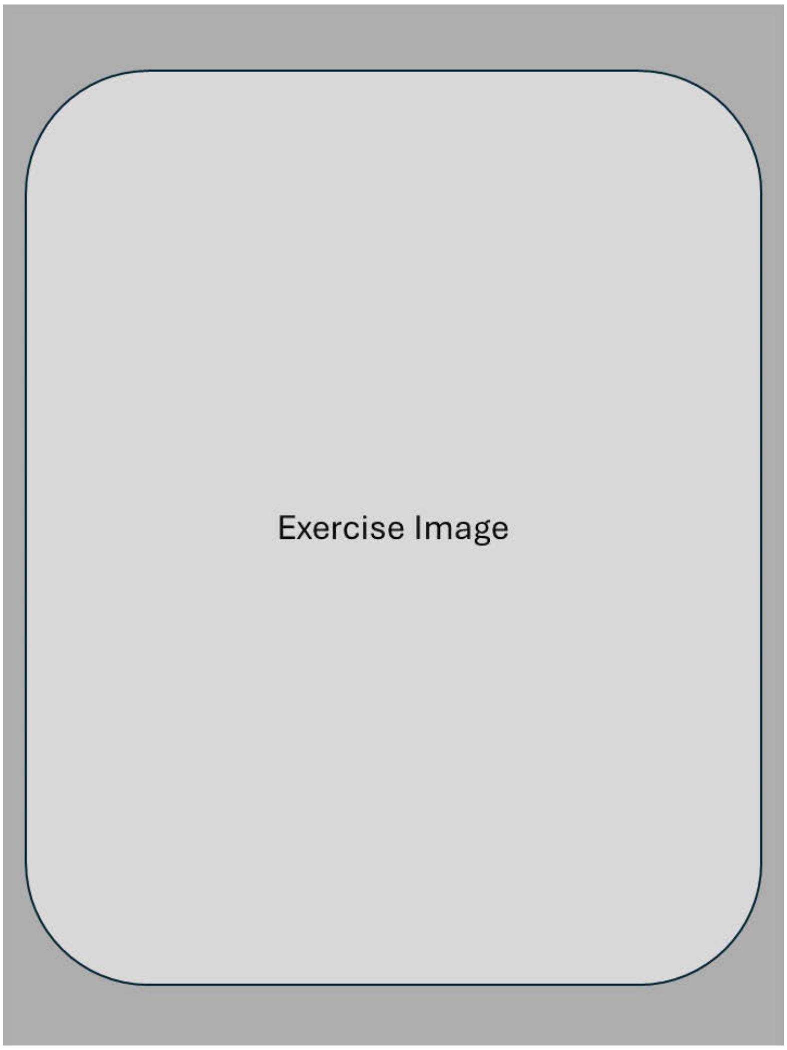

Running/Jogging on a Treadmill

Step onto the treadmill and attach the safety key to your clothing.

Turn on the treadmill and start walking. Begin at low intensity and add speed gradually until you reach the speed of your choice. Gradually lower the speed before stopping.

Continue for 15 min .

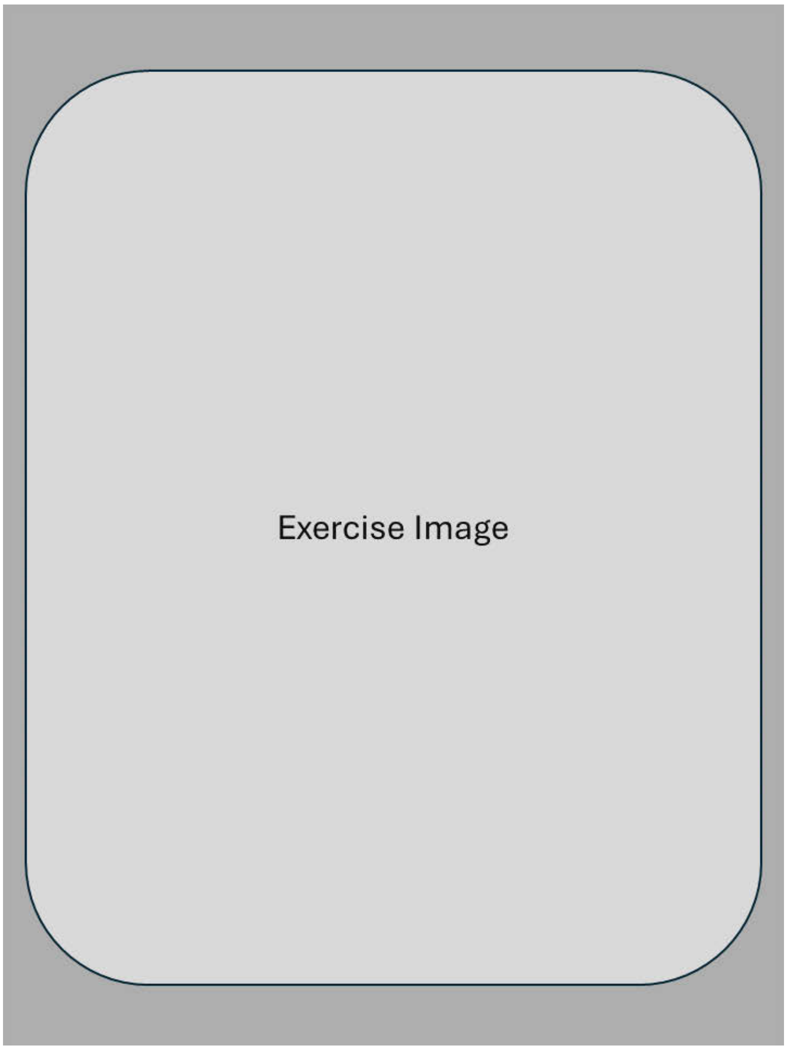

Knee Extension in a Machine

Sit up straight on a knee extension machine with your knees bent and the resistance lever in front of your ankles.

Straighten your knees. In a controlled manner let your knees bend back to the starting position. Do the knee movement 0-30 grade angle.

Repeat 12 times. Do 3 sets

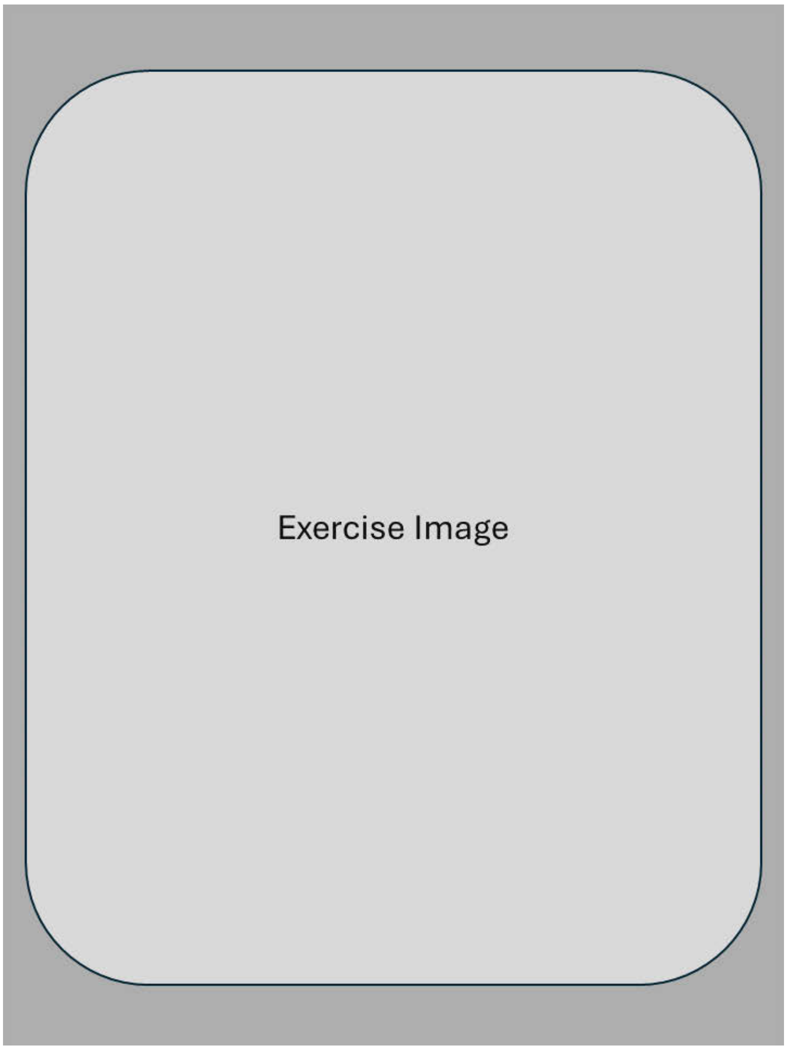

Seated Leg Curl

Sit up straight on a leg curl machine with your knees straight and the resistance lever behind your ankles.

Bend your knees and bring heels towards your buttocks. In a controlled manner let your knees straighten back to the starting position.

Note: Some machines also have another support that should be on the front side, just under your knees.

Repeat 12 times. Do the

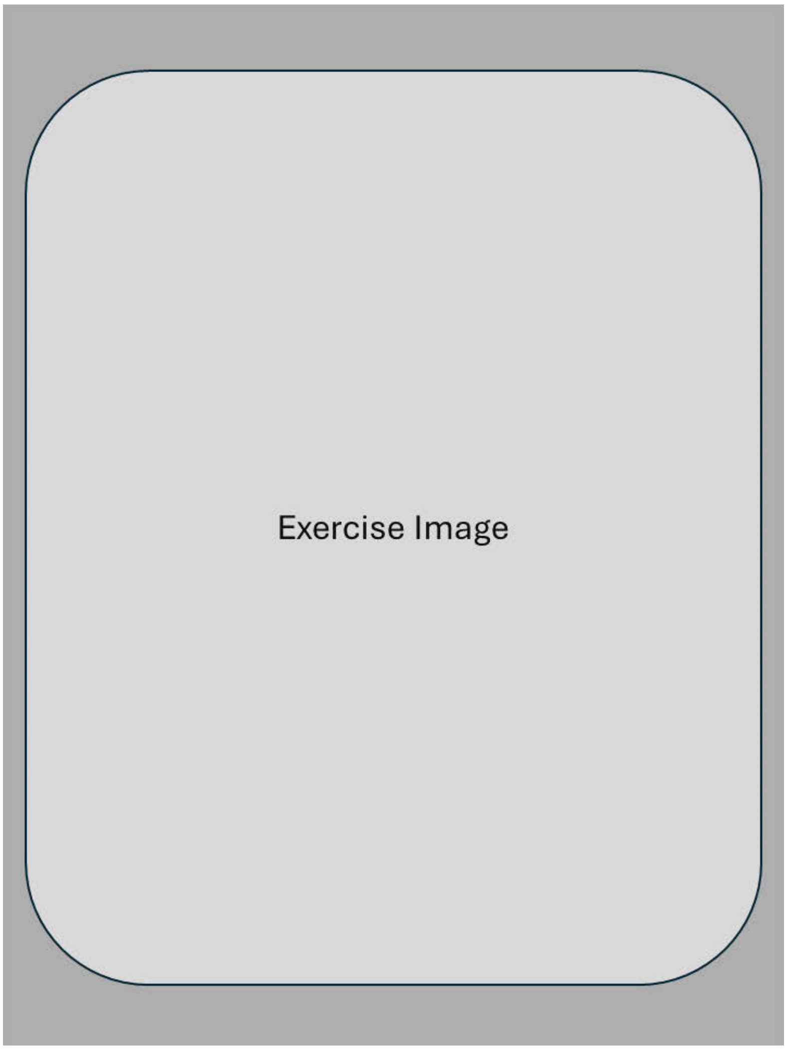

Split Squat with Dumbbells

Stand tall with feet hip-width apart and one foot in front of the other. Hold dumbbells in both hands.

Squat down and keep your trunk in an upright position. Push back up and straighten your hips.

Note:
- Keep hips, knees and toes aligned.
- Keep your weight evenly distributed between your forefoot and heel.

Repeat 12 times. Do 3 sets

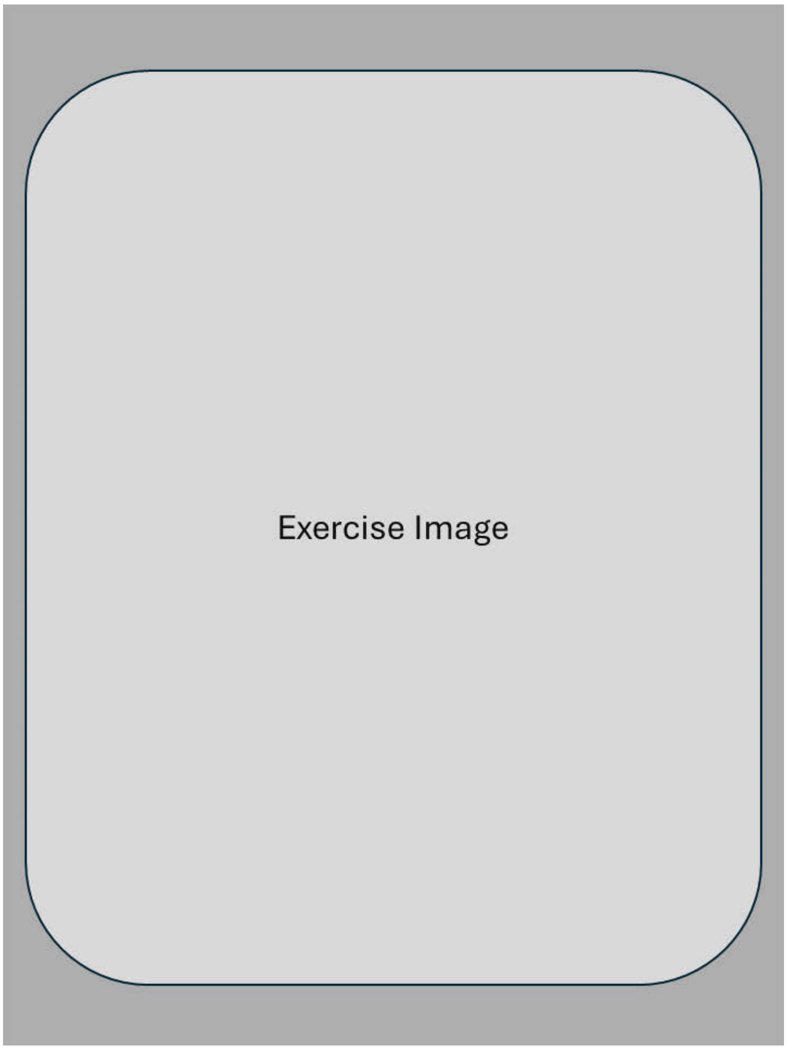

Single-leg Deadlift

Stand tall on one leg, holding a weight plate near your trunk in front of you with bent arms.

Lean forward with your trunk bending forward from the hip with the knee of the standing leg slightly bent. Keep your spine in neutral position and straighten the other leg backwards (into straight line with your body). Simultaneously lower the weight towards the floor to straight arms.

Use your hamstrings and buttock muscles to return to the starting position while maintaining the straight line of the rear leg and trunk.

From single-leg standing position continue by pressing the weight up to straight arms into an overhead position, rise onto your toes and lift knee high. Find your balance and then return to the starting position.

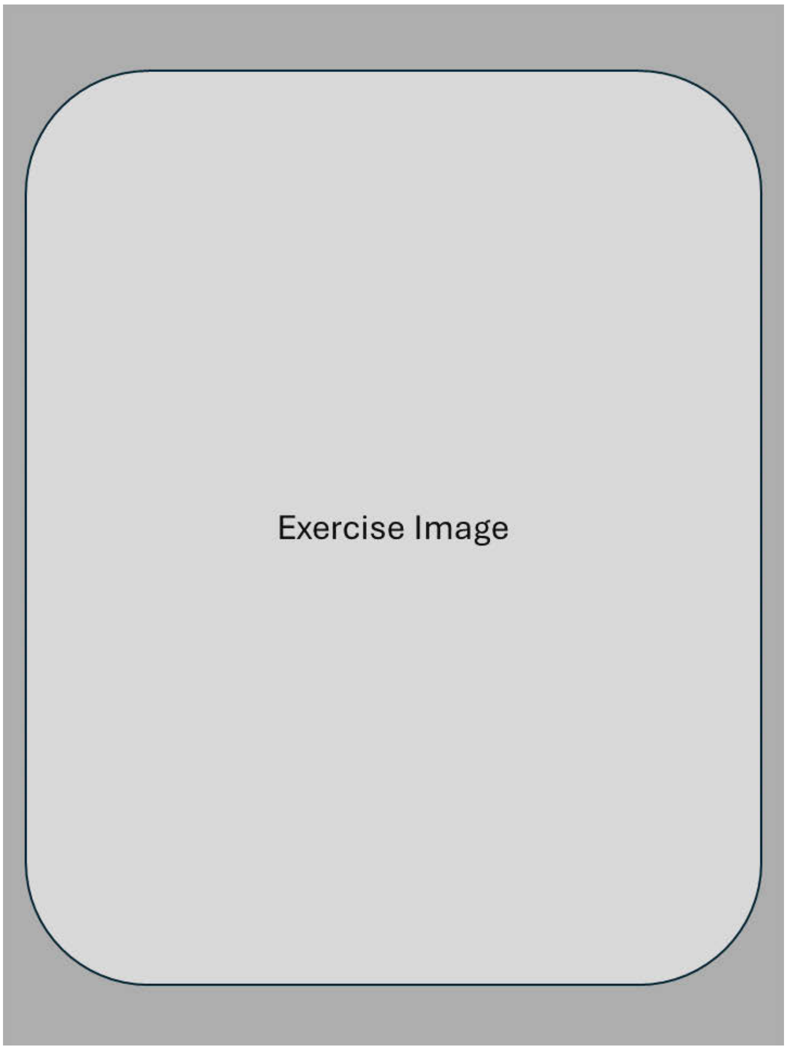

Use your hamstrings and buttock muscles to return to the starting position while maintaining the straight line of the rear leg and trunk.

From single-leg standing position continue by pressing the weight up to straight arms into an overhead position, rise onto your toes and lift knee high. Find your balance and then return to the starting position. Repeat 12 times. Do 3 sets

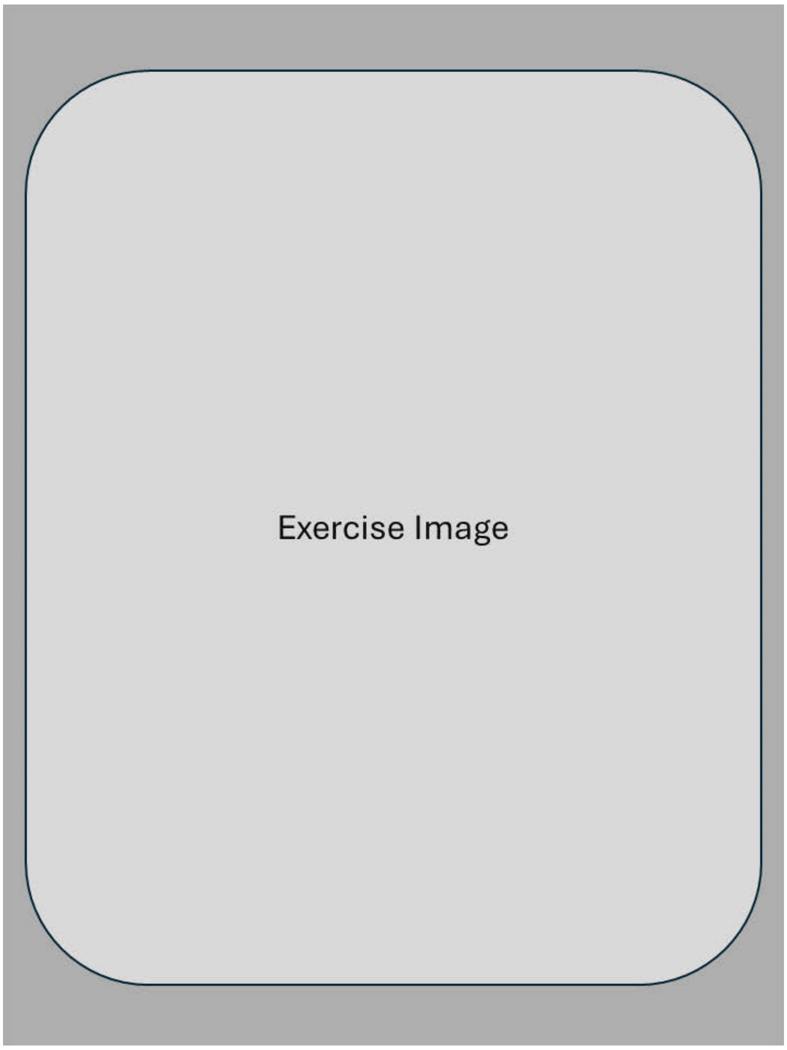

Back Squat

Stand with your feet hip-width apart. A bar is placed on top of posterior shoulder muscles and trapezius (low position) or above posterior shoulder muscles at the base of the neck (high position). Shoulders are pinned back and down. Hands are just outside shoulders or slightly wider.

Squat down by moving hips backwards and down. Keep weight evenly on whole foot, knees aligned with toes and your chest up. Push back up and fully straighten your hips and knees.

Note: Don’t let your upper body bend excessively forwards. Repeat 12 times. Do 3 sets

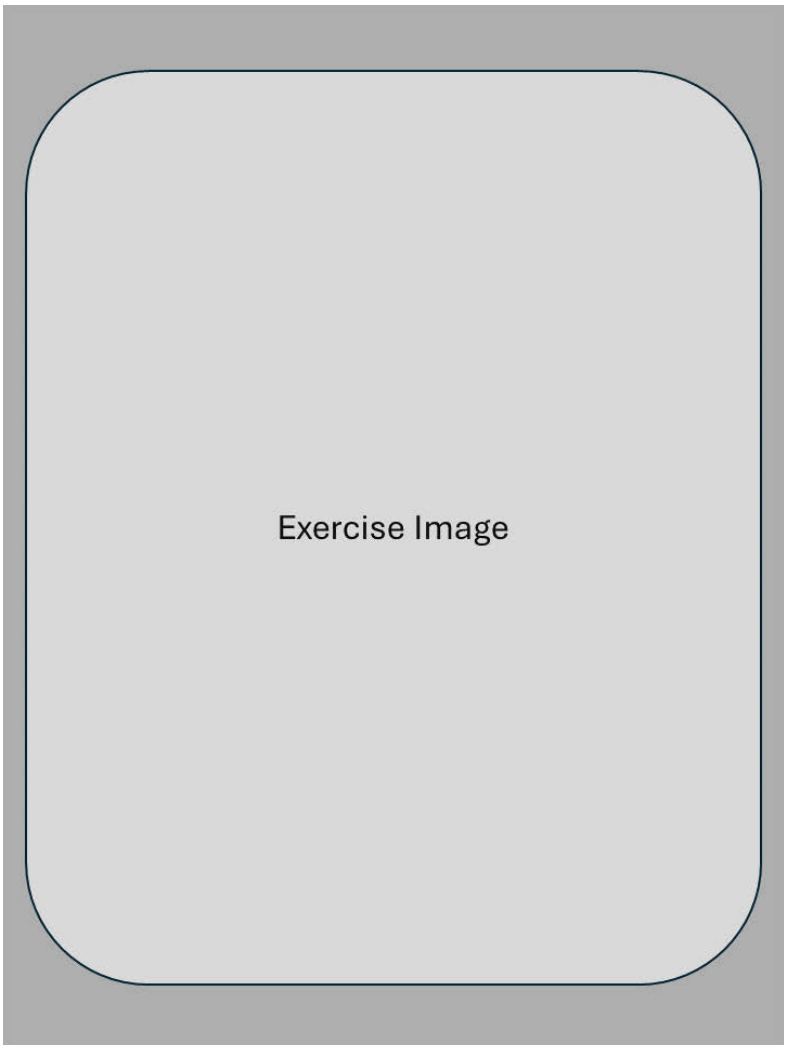

Leg Press

Sit up straight on a leg press machine, with your feet placed hip-width apart on the platform. Release the safety locks and lower down to a squat and lock the position.

Push through your heels and straighten your knees. Actively push your knees out, to avoid them collapsing in. In a controlled manner lower yourself back to the squat.

Note: The weight is evenly distributed between the heels and forefeet.

Repeat ___12___ times. Do 3 sets

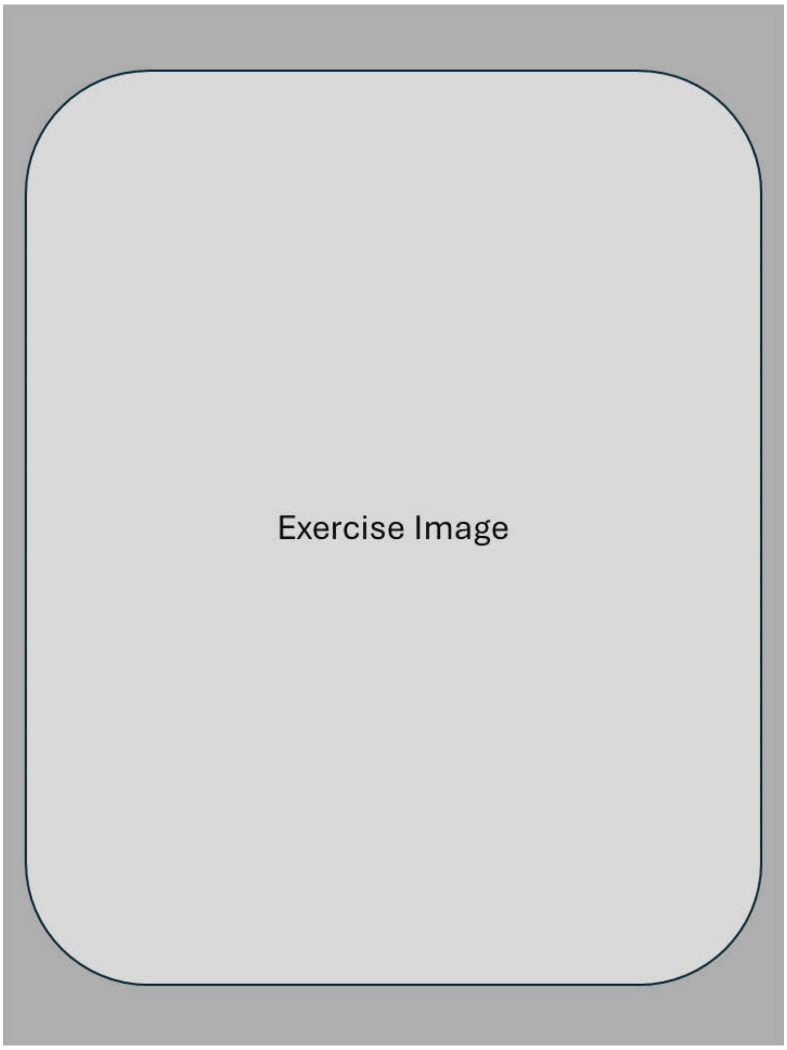

Inclined Single-leg Leg Press

Sit up straight on a leg press, with one foot on the floor and the other foot placed on the platform. Release the safety locks.

In a controlled manner let your knee and hip bend, until your knee is close to 90 degrees. Push the platform away and straighten your knees. When you get off the leg press, remember the safety locks.

Note:
- Keep your knee aligned with your toes.
- The weight is evenly distributed between the heel and forefoot.

Repeat 12 times. Do 3 sets

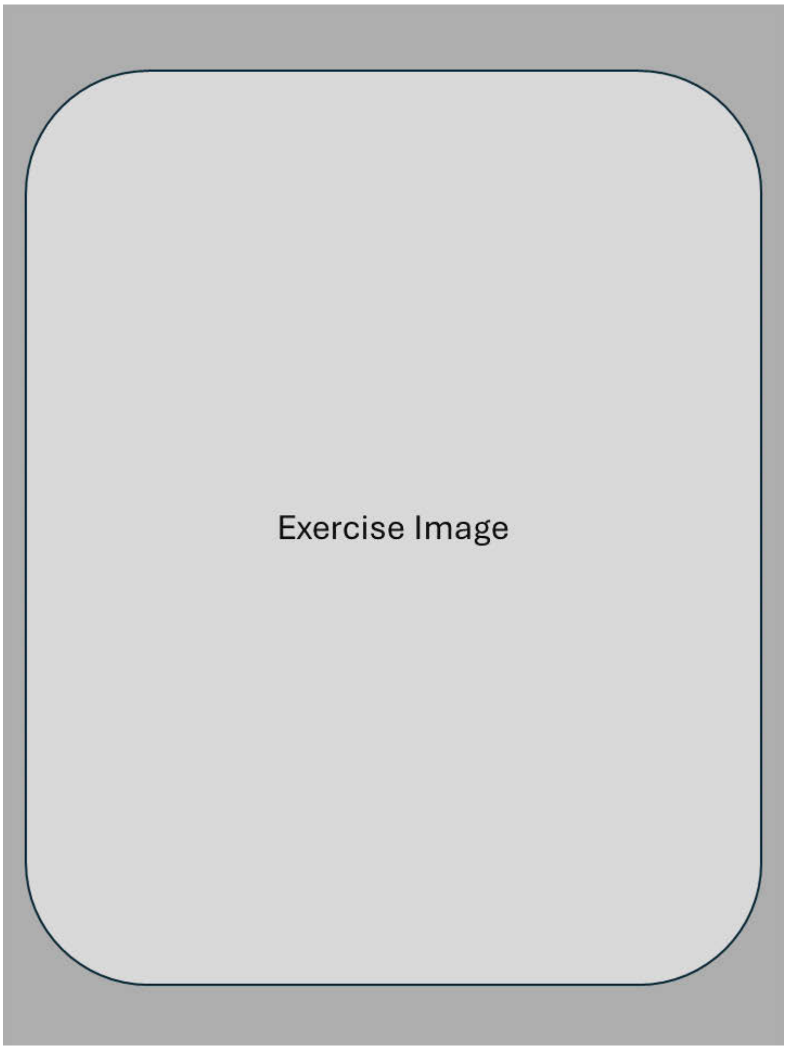

Calf Press

Sit up straight on a leg press machine with the balls of your feet placed near the lower edge of the leg press platform with the heels hanging off.

With your knees straight, press against the platform and straighten your ankles. In a controlled manner let your ankles bend, while keeping your knees straight.

Repeat 12 times. Do 3 sets
