# Supplementary material for: Effect of accelerated postoperative rehabilitation after tibial tubercle distalisation: A randomised controlled trial protocol
Source: PLoS One. 2024 Jul 11;19(7):e0304075. doi: 10.1371/journal.pone.0304075 (PMC11239065; doi:10.1371/journal.pone.0304075)
Supplement: S10 File — (DOCX) [file pone.0304075.s010.docx]

# Effect of accelerated postoperative rehabilitation after tibial tubercle distalization: a randomized controlled trial

# Nopeutetun leikkauksen jälkeisen kuntoutuksen vaikutus sääriluun kyhmyn siirron jälkeen: satunnaistettu kontrolloitu tutkimus

Suomenkielinen yhteenveto v1.3

Timo Rahnel¹, Frederick K. Weitz², Ville M. Mattila ⁴, Antti P. Launonen ⁴, Aleksi Reito^4^, Erkki Nilkku³, Petri J. Sillanpää²

1: Dept. of Orthopedic surgery, North Estonia Medical Centre, 19 J. Sütiste Str

13419 Tallinn, Estonia

2: Pihlajalinna, Koskisairaala Hospital Hatanpään valtatie 1, 33100 Tampere Finland

3: Pihlajalinna, Koskiklinikka physiotherapy department Hatanpään valtatie 1, 33100 Tampere Finland

4: Dept. of Orthopedic surgery, University of Tampere, Teiskontie 35, 33521 Tampere, Finland

1. **Yhteenveto**

Tutkimuksen tarkoitus on selvittää kahden eri postoperatiivisen kuntoutusprotokollan eroja. Toinen protokolla on yleisesti käytössä ollut rajoittava protokolla ja toinen ns. nopean kuormituksen protokolla. Potilaat randomoidaan kahteen ryhmään protokollan mukaan. Post operatiivisesti seurataan reisilihasvoiman kehittymistä ja liikealan palautumista. Kontrollit ovat 6,12,24 ja 52 viikkoa leikkauksesta

1. **Johdanto**

Patella alta on polvilumpion korkeusaseman variaatio, missä polvilumpio sijaitsee anatomisesti liian proksimaalisesti. Tässä tilanteessa polvilumpion ja troklean rustopinnoilla on joko huono kontakti tai ei lainkaan kontaktia. Tämä anatominen variaatio saattaa aiheuttaa polvilumpion epävakautta ja tästä johtuvaa toistuvaa polvilumpion luksoitumista (sijoiltaanmeno). Oireena saattaa myös olla rasituskipu, ilman polvilumpion epävakautta.

Polvilumpion poikkeavan korkea asema (patella alta) on yksi merkittävä polvilumpion sijoiltaan menolle ja polven etuosan kivulle altistava tekijä. Polvilumpion korkeusaseman mittaamiseen käytetään useampia indeksejä ja tieteellisessä kirjallisuudessa ei ole yksiselitteistä määritelmää siitä, mikä on liian korkealla oleva polvilumpio.([1](#_ENREF_1)) Indekseistä käytetyimmät ovat Insall-Salvati, Caton-Dechamps ja Blacburne-Peel([2-5](#_ENREF_2)). Nämä indeksit mittaavat polvilumpion mittaa suhteessa sääriluun kyhmyyn (tuberositas tibiae) tai sääriluun anterioriseen kulmaan keskilinjassa. Indeksit ovat suuntaa antavia. Patelotrokleraarinen(PTI) indeksi on tuorein ja antaa parhaan käsityksen rustopintojen kontaktista polvilumpion ja reisiluun troklean välillä([1](#_ENREF_1)). PTI muuttuu polvikulman muuttuessa ja tämän vuoksi tämäkään indeksi ei ole täysin luotettava([6](#_ENREF_6)).

Tuberositas tibiaen distalisaatio osteotomia (DTTO) on polvilumpion poikkeavan korkeusaseman (Patella alta) korjaamiseksi sovelias leikkaushoito ([7](#_ENREF_7), [8](#_ENREF_8)). Yleisesti DTTO:n jälkikuntoutuksessa on käytetty ortoosia rajoittamaan polven koukistusta varhaisessa toipumisvaiheessa, lisäksi raajan kuormitusta on rajoitettu. Ortoosia on pidetty 4-8 viikkoa ja kuormitusrajoitukset ovat olleet 4-6 vko mittaiset. Tässä protokollassa on potentiaalisia riskejä polven liikeala ongelmalle ja reisilihaksen voimaheikkoudelle mikä pitkittää toipumista ja saattaa aiheuttaa tarvetta toimenpiteille.

1. **Tavoitteet**

Tutkimuksen tavoitteena on verrata kahden erilaisen leikkauksen jälkikuntoutusohjelman eroja. Primaari päätemuuttuja on liikeala (ROM) 3kk kuluttua leikkauksesta, missä merkitsevänä erona pidetään 10 astetta.

Tutkimus vastaa myös siihen, että syntyykö rajoitetun ja vapaan kuormituksen kuntoutusprotokollien välillä eroja välittömiin leikkauksen jälkeisiin (Fiksaation pettäminen, rasitusmurtuma) ja minkälainen ero on voiman palautumisessa.

**Tutkimusaineisto, menetelmät ja luvat**

Tutkimukseen rekrytoidaan kaikki kasvunsa päättäneet 15-35-vuotiaat potilaat, joille tehdään tuberositas tibiaen (TT) distalisaatio (NGL66) Koskisairaalassa tutkimusryhmän jäsenten (Petri Sillanpää, Frederick Weitz) toimesta.

*Tarkoituksena on, että tutkimusrekrytointi, toimenpiteet, sekä kontrollit suoritetaan Koskiklinikassa em. Kirurgien toimesta. TAYS toimii tutkimuksen akateemisena osapuolena, sekä ohjausvastuussa LL Timo Rahnelin väitöskirjan ohjauksesta.*

Sopivan potilaan tullessa vastaanotolle hänelle kerrotaan tutkimuksesta, annetaan kirjallinen tiedote, sekä kerrotaan suullisesti tutkimuksen kulusta. Mikäli potilaalla on kysymyksiä, näihin vastataan. Mikäli potilas haluaa osallistua tutkimukseen (satunnaistettuun ryhmään) tai seurantaryhmään, hän antaa tietoisen suostumuksen osallistumisesta.

Tutkimussuunnitelmaan sisältyy preoperatiivinen arvio polven toiminnasta (Kujala/Banf score ja KOOS), postoperatiiviset kontrollit 6, 12 ja 52 viikkoa toimenpiteestä. Osteotomian luutumista arvioidaan röntgenkuvalla 6 ja 12 viikkoa toimenpiteestä. Lääkärin kontrollit viikolla 6 ja 12. Kyselyt suoritetaan ennen leikkausta ja viikoilla 6, 12, 24 ja 52 leikkauksen jälkeen. Tutkimusfysioterapeutin kontrollit ovat viikoilla 6, 12, 24 ja 52. Fysioterapia protokollan kesto on 24 viikkoa (liite protokolla 1 ja protokolla 2). Fysioterapia kontrollissa mitataan polven liikeala ja voimantuotto fleksio- ja ekstensiosuuntaan (koukistus ja ojennus).

Tutkimukseen rekrytoidaan 63 potilasta molempiin ryhmiin, yhteensä 126 potilasta. Tutkimukseen osallistuminen perustuu vapaaehtoisuuteen. Potilaat allekirjoittavat suostumuskaavakkeen ja alaikäisten potilaiden kohdalla suostumuskaavakkeen allekirjoittavat sekä potilas, että tämän huoltaja. Tutkimukseen hyväksytään vain potilaat, kenellä tuberositas tibian distalisaatio on itsenäisenä toimenpiteenä, tai liitännäistoimenpiteenä on MPFL rekonstruktio gracilis-siirteellä.

1. **Aikataulu**

Tutkimusluvat haetaan keväällä 2022, Aineiston keruu on suunniteltu alkavaksi syksyllä 2022. Kyseessä on lyhyen aikavälin paranemista dokumentoiva tutkimus, jonka suunniteltu kesto on noin 3 vuotta. Aineiston keruu (leikkaukset) vuoden 2025 loppuun mennessä. Jälkikontrollit vuoden 2026 loppuun mennessä sekä sen jälkeen aineiston analysointi ja julkaiseminen.

1. **Eettiset näkökohdat**

Kyseessä on satunnaistettu kontrolloitu tutkimus, jossa tutkittavat satunnaistetaan kahteen eri interventio ryhmään. Tutkittavia pyydetään täyttämään kyselykaavakkeet. Osallistuminen on vapaaehtoista ja tutkittavalla on oikeus milloin tahansa perua osallistumisensa tutkimukseen. Sairaskertomustietoihin, kuvantamistutkimuksiin sekä kyselyn vastauksiin perustuen luodaan tutkimuksen ajaksi tutkimusrekisteri. Elektroninen aineisto säilytetään Tampereen Yliopiston RedCap-tutkimuspalvelimelle. Paperiset aineistot säilytetään pseudonymimuodossa tutkimuskeskuksessa (Koskiklinikalla) ja paperiaineisto siirretään elektroniselle käsin tai suoraan potilaan syöttämänä. Lopullinen pseudonymimuodossa oleva aineisto analysoidaan Virossa Tallinnassa päätutkijan toimesta. Aineisto toimitetaan salattuna tiedostona suojasähköpostia käyttäen.

Tutkimusryhmän jäsenten on tutkimuksen aikana lisäksi mahdollista käyttää salasanalla suojattua rekisteritiedostoa tiedon analysointiin. Mahdollinen syntyvä manuaalinen aineisto hävitetään tietosuojaohjeistuksen mukaisesti 5 vuotta tutkimuksen päättymisen jälkeen. Tutkimuksen päätyttyä tutkimusrekisteri säilytetään viiden vuoden ajan Tampereen Yliopiston RedCap tutkimuspalvelimelle, minkä jälkeen se hävitetään. Kaikkia tutkimusryhmän jäseniä koskee vaitiolovelvollisuus tunnisteellisten potilastietojen osalta. Tutkimustulokset julkaistaan sellaisessa muodossa, ettei yksittäistä tutkimukseen osallistunutta henkilöä voida tunnistaa.

1. **Rahoitus**

Tutkimuksella ei ole rahoitusta.

1. **Sidonnaisuudet**

Tutkimusryhmän jäsenillä ei ole aiheeseen liittyviä sidonnaisuuksia.

1. Biedert RM, Tscholl PM. Patella Alta: A Comprehensive Review of Current Knowledge. American journal of orthopedics. 2017 Nov/Dec;46(6):290-300. PubMed PMID: 29309446.

2. Insall J, Salvati E. Patella position in the normal knee joint. Radiology. 1971 Oct;101(1):101-4. PubMed PMID: 5111961.

3. Caton J. [Method of measuring the height of the patella]. Acta orthopaedica Belgica. 1989;55(3):385-6. PubMed PMID: 2603679. Methode de mesure de la hauteur de la rotule.

4. Caton J, Deschamps G, Chambat P, Lerat JL, Dejour H. [Patella infera. Apropos of 128 cases]. Revue de chirurgie orthopedique et reparatrice de l'appareil moteur. 1982;68(5):317-25. PubMed PMID: 6216535. Les rotules basses. A propos de 128 observations.

5. Caton J, Mironneau A, Walch G, Levigne C, Michel CR. [Idiopathic high patella in adolescents. Apropos of 61 surgical cases]. Revue de chirurgie orthopedique et reparatrice de l'appareil moteur. 1990;76(4):253-60. PubMed PMID: 2148403. La rotule haute idiopathique chez l'adolescent. A propos de 61 cas operes.

6. Ahmad M, Janardhan S, Amerasekera S, Nightingale P, Ashraf T, Choudhary S. Reliability of patellotrochlear index in patellar height assessment on MRI-correction for variation due to change in knee flexion. Skeletal radiology. 2019 Mar;48(3):387-93. PubMed PMID: 30141067.

7. Ambra LF, Phan A, Gomoll AH. A New Technique for Distalization of the Tibial Tubercle That Allows Preservation of the Proximal Buttress. Orthopaedic journal of sports medicine. 2018 Sep;6(9):2325967118798621. PubMed PMID: 30263901. Pubmed Central PMCID: 6156213.

8. Sherman SL, Erickson BJ, Cvetanovich GL, Chalmers PN, Farr J, 2nd, Bach BR, Jr., et al. Tibial Tuberosity Osteotomy: Indications, Techniques, and Outcomes. The American journal of sports medicine. 2014 Aug;42(8):2006-17. PubMed PMID: 24197613.
