# Supplementary material for: Effect of accelerated postoperative rehabilitation after tibial tubercle distalisation: A randomised controlled trial protocol
Source: PLoS One. 2024 Jul 11;19(7):e0304075. doi: 10.1371/journal.pone.0304075 (PMC11239065; doi:10.1371/journal.pone.0304075)
Supplement: S11 File — (DOCX) [file pone.0304075.s011.docx]

# Effect of accelerated postoperative rehabilitation after tibial tubercle distalisation: A randomised controlled trial

Timo Rahnel¹, Frederick K. Weitz², Ville M. Mattila ⁴, Antti P. Launonen ⁴, Aleksi Reito^4^, Erkki Nilkku³, Petri J. Sillanpää²

1: Dept. of Orthopaedic Surgery, North Estonia Medical Centre, 19 J. Sütiste Str

13419 Tallinn, Estonia

2: Pihlajalinna, Koskisairaala Hospital, Hatanpään valtatie 1, 33100 Tampere, Finland

3: Dept. of Physiotherapy, Pihlajalinna, Koskiklinikka, Hatanpään valtatie 1, 33100 Tampere, Finland

4: Dept. of Orthopaedic Surgery, Tampere University Hospital, Teiskontie 35, 33521 Tampere, Finland

**Corresponding Author:**

Timo Rahnel

North Estonia Medical Centre, Sütiste tee 19, 13419 Tallinn, Estonia

Phone:

E-mail: [timo.rahnel@regionaalhaigla.ee](mailto:timo.rahnel@regionaalhaigla.ee)

**Abstract**

Patella alta is a clinical condition where the patellar is positioned too proximal in relation to the femoral trochlea. Such an abnormality may cause patellar instability and predispose to recurrent patellofemoral dislocations and patellofemoral pain. There are no conclusive guidelines for determining a threshold for too high positioned patella, as several different methods have been described to measure patellar height. As a surgical solution, distalising tibial tubercle osteotomy (DTTO) has been described to correct excessive patellar height.

In the early phase of the DTTO postoperative protocol, weightbearing and knee flexion are limited with a brace commonly for 4-8 weeks. The potential risks for adverse effects associated with the limitation rehabilitation protocol include a delay in regaining knee range of motion (ROM), stiffness and muscle weakness. As a result, recovery from surgery is delayed and may lead to additional procedures and long-term morbidity in knee function.

This is a prospective, randomised, controlled, single-blinded, single centre trial comparing a novel accelerated rehabilitation protocol with the traditional, motion restricting rehabilitation protocol. All skeletally mature patients aged 15-35 years, referred to as the DTTO procedure group, are eligible for inclusion in the study. Patients will be randomised to either the fast rehabilitation group or the traditional rehabilitation group. Patients with patellar instability will be additionally treated with MPFL reconstruction.

The hypothesis of the trial is that the novel accelerated rehabilitation protocol will lead to faster recovery and improved functional outcome at 6, 12 and 24 weeks compared with the conservative rehabilitation protocol. A secondary hypothesis is that the complication rate will be similar in both groups.

The study will document short-term recovery and the planned follow-up will be 3 years. After the 1-year follow-up, the trial results will be disseminated in a major peer-reviewed orthopaedic publication.

**Introduction**

The term patella alta refers to the abnormal height of the patellar, where the anatomical position of the patellar is too proximal. In cases of patella alta, patellar and trochlear cartilage surfaces have limited or no contact. This anatomical variation can cause patellar instability and result in recurrent patellofemoral dislocations. One of the possible symptoms caused by patella alta can be patellofemoral pain with or without patellar instability. In addition, patella alta is a significant risk-factor for patellofemoral dislocations and anterior knee pain.

At present, there are no conclusive guidelines to determine a patella that is positioned too high, as several methods have been described to measure patellar height in the scientific literature [[2](#_ENREF_2)]. Methods that use bony tibial landmarks, such as the Insall-Salvati index [[5](#_ENREF_5)], the Blackburne-Peel index and the Caton-Deschamps index [[3](#_ENREF_3), [4](#_ENREF_4)], are the most commonly used methods to measure patellar height. These indices compare patellar length to the distance to the tibial tubercle or the tibial anterior superior edge in the midline. The patellotrochlear index (PTI) is a novel measure to concretise the contact area between the patellar and trochlear joint surfaces. [1] However, since the PTI value changes with different knee flexion angles, the index is unreliable. [6].

Distalising tibial tubercle osteotomy (DTTO) is a suitable surgical procedure to treat patella alta. [[1](#_ENREF_1), [9](#_ENREF_9)]. During the early rehabilitation phase after DTTO, it is common to limit weightbearing and knee flexion with orthosis. In general, the orthosis is used for between 4 and 8 weeks and limited weightbearing is maintained for 4 to 6 weeks. The potential risks of this rehabilitation protocol are restricted knee ROM and muscle weakness, leading to delayed recovery and the possible need for additional procedures.

In our previous, as yet unpublished, retrospective study, we have shown that accelerated rehabilitation is a feasible treatment method after DTTO, and we believe that patient aftercare can be more aggressive than at present. However, to the best of our knowledge, no high-quality randomised controlled trials have been conducted to date on postoperative rehabilitation protocols after DTTO.

Thus, the aim of this randomised controlled trial (RCT) is to identify the differences between the results of two postoperative rehabilitation protocols. One of the protocols is a typical conservative rehabilitation protocol and other is a novel accelerated rehabilitation protocol.

**Materials and methods**

In this is prospective, randomised, controlled, single-blinded, single centre trial, we compare an accelerated rehabilitation protocol with a typical conservative rehabilitation protocol after DTTO. The trial has been reported in accordance with the Consolidated Standards of Reporting Trials (CONSORT) guidelines. Figure 1 and Table 1

Figure 1 Study flow chart

Recruitment

Data collection and analysis

KOOs score, Banff patella instability score, Range of motion, muscle strength test

Outcome measurement at 6, 12, 24 and 52 weeks

Conservative rehabilitation protocol

Aggressive rehabilitation protocol

Randomisation 1:1

Baseline asessement

Informed consent

Screening

Table 1 Study design and assessments

|  |  |  | Study period | |  |  |
| --- | --- | --- | --- | --- | --- | --- |
|  | Enrolment | Allocation | Post allocation | |  |  |
| Study visit | 1 | 2 | 3 | 4 | 5 | 6 |
| Timepoint | Preoperative | Surgery day (0) | 6 weeks | 12 weeks | 24 weeks | 52 weeks |
| **Enrolment:** |  |  |  |  |  |  |
| Eligibility screening | x |  |  |  |  |  |
| Informed consent | x |  |  |  |  |  |
| Demographics and medical data | x |  |  |  |  |  |
| Allocation |  | x |  |  |  |  |
| **Intervention** |  |  |  |  |  |  |
| TT-distalisation |  | x |  |  |  |  |
| **Assessments** |  |  |  |  |  |  |
| BPII score | x |  | x | x | x | x |
| KOOS Score | x |  | x | x | x | x |
| Adverse events |  | x | x | x | x | x |

The hypothesis of the trial is that the novel accelerated rehabilitation protocol will lead to faster recovery and improved functional outcome at 6, 12 and 24 weeks compared with the conservative rehabilitation protocol. The secondary hypothesis is that the complication rate will be similar for both protocols.

The primary outcome in this study will be knee range of movement (ROM) measured at 12 weeks postoperatively. A difference of 10º in full range of movement will be considered significant. The results will be measured with a long goniometer in a standardised manner. Secondary outcomes will be knee ROM measured at baseline, 6, 24 and 52 weeks postoperatively, the Knee Injury and Osteoarthritis Outcome Score (KOOS) and the Banff Patella Instability Instrument (BPII) score measured at baseline, 6, 12, 24 and 52 weeks, and isometric muscle strength measured at baseline, 12, 24 and 52 weeks postoperatively. Regained isometric muscle strength will be measured during follow-up visits and calculated to a ratio compared with a preoperative value. The number of reoperations and complications (failure of fixation and stress-fractures) will be also reported as secondary outcomes. The KOOS questionnaire is an instrument to assess patients’ opinions about their knees and any associated problems. KOOS consists of 5 subscales: Pain, other Symptoms, Function in daily living (ADL), Function in sport and recreation (Sport/Rec) and knee related Quality of life (QOL). BPII is also a patient-reported outcome measurement instrument. BPII is, however, more specific for patellofemoral problems.

After enrolment, all the patients visit the study physiotherapist. The baseline scores from ROM, isometric muscle strength, KOOS and BPII are recorded during the visit. Randomisation to the allocation groups will be done after surgery.

The ROM measurements are taken with a goniometer, with the patient in supine position. The active range of motion is measured as the patient moves the knee in a full range of active flexion and extension. Range of motion is then measured. Passive range of motion is measured in the same supine position. The examiner first moves the knee in a full range of flexion and then in a full range of extension. Range of motion is then measured. Full range of movement is 5º-0-140º. A range of movement of 0º-130º is considered adequate.

Isometric muscle strength is measured in sitting position. The measurement is taken with a dynamometer at three different knee angles 5°, 39° and 90°.

KOOS and BPI are taken before the muscle strength and ROM measurements.

## Patient selection

The eligible study population will comprise all consecutive patients aged 15 to 35 years with closed growth plate (physis) who have been referred for DTTO surgery at Pihlajalinna Koskisairaala Hospital, Tampere, Finland. The upper age limit was chosen to minimize the effect of patellofemoral osteoarthritis. As the DTTO procedure can only be planned for skeletally mature patients, the closure of the physis will be evaluated with magnetic resonance imaging (MRI) and plain radiographs. The Greulich and Pyle (GP) method will be used to define skeletal age if needed.[[8](#_ENREF_8)]

Only those patients with isolated DTTO or DTTO combined with medial patellofemoral ligament (MPFL) reconstruction will be included in the study.

The following criteria will be used for patient selection throughout the study.

Inclusion criteria:

- Symptomatic patella alta with recurrent dislocation or subluxation. Long-lasting anterior knee pain not responding to rehabilitation

Exclusion criteria:

Radiographic

- Open growth plates
- Iwano [[7](#_ENREF_7)] grade III and IV changes in patellofemoral joint
- Caton-Deschamps <1.1 in MRI
- PTI >50% in MRI
- High grade trochlear dysplasia

General

- Refuses to participate in the study
- Aged less than 15 or more than 35 years
- Severe neurological, pulmonal or cardiovascular comorbidities that are contraindications for surgery
- Lack of adequate co-operation
- Does not adequately understand written and spoken instructions in the local language

Those patients who decline to take part in the trial will be asked to join a follow-up cohort as a background population. The patients in this follow-up cohort will be treated “as normal” without allocation, but the follow-up questionnaires will be the same as those given to the randomly assigned population. The patients in the follow-up cohort will also be asked to provide informed written consent.

## Randomisation

Patients will be randomised using a pre-trial random number matrix in block allocation fashion in blocks of 10. The blocks will be stratified by age (under 20 and 20-35 years) since age has been shown to associate with the main outcome measure. The Caton-Deschamps index will also be used for stratification. Randomisation will be performed using an online randomisation platform (Redcap). After informed written consent has been received and the intervention in the OR performed, the patients will be randomly allocated for one of the rehabilitation protocols. The physician responsible for the intervention or treatment will not participate in the collection of patient outcomes during the follow-up. The research coordinator will monitor the study flow. An independent monitoring committee, which was established during our previous RCT, will monitor the study.

## Surgical treatment

Operative treatment will be performed by trained and experienced knee surgeons. The surgeons’ skills and number of procedures will be reported according to the criteria given by the Consort Group.[[10](#_ENREF_10)]

The aim of the surgical treatment is to normalise the biomechanics of the patellofemoral joint. The operation will start with an arthroscopic examination to verify patella alta position. The high-positioned patella in relation to the trochlea will be documented and an approximation made of the required distalisation for adequate cartilage contact between the patella and the trochlea. Thereafter, a 5-7 cm mid-line incision will be made at the tibial tubercle. The gracilis graft will be harvested from the same incision if needed for MPFL-reconstruction. The lateral aspect of the tibial tubercle will be exposed. The proximal distalisation cut will be made in AP direction at 70º angle at a minimum of 6-7 cm distally from the tibial tubercle. The distal AP cut will be done according to preoperative measurements taken from imagining data and the arthroscopic findings of the patient. The bone block will be removed to allow distalisation of the tubercle. Furthermore, the tubercle will be mobilised with chisels to allow the distalisation of the tubercle. The osteotomy will be fixed with two 4.5 mm AO screws. The appropriate localisation of the fixation and patella will be confirmed with fluoroscopy.

## Rehabilitation

Patients will be randomised in two rehabilitation groups. Patients in both groups will be guided by in-ward physiotherapists and will be given written physiotherapy guidelines for both instructed physiotherapy and self-guided exercises. After discharge from the hospital, patients will be referred to physiotherapy for further guidance. Both groups will start preliminary exercises from the first postoperative day to reduce oedema in the operated lower limb. For the detailed rehabilitation guidelines, please see Table 2.

**Table 2**

**The rehabilitation guidelines**

| Elements of physical therapy | Group 1 Accelerated rehabilitation | Group 2 Conservative rehabilitation |
| --- | --- | --- |
| Antioedema knee, calf, leg | Day 1 | Day 1 |
| Knee brace, ROM limitations | No | 6 weeks |
| Weight-bearing limitations | No, Crutches are recommended for 4 weeks. | 4 weeks limb weight,  followed by half body weight till week 6 |
|  |  |  |
| Active exercises. Functional exercises. | 1 week | 8 weeks |
| Active dynamic strengthening exercises. | 5 weeks | 8 weeks |
| Closed chain exercises for muscle strengthening. | 8 weeks | 8 weeks |
|  |  |  |
| Muscle endurance and neuromuscular control, progress strengthening exercises, jogging | 12 weeks | 12 weeks |

## Follow-up

Postoperative physiotherapy control visits will take place at 6, 12, 24 and 52 weeks after the procedure. The physiotherapy control visits will be managed by a senior physiotherapist who will be blinded to the allocation group. During the visits, knee ROM and isometric flexion/extension strength will be measured. The isometric extension and flexion strength will be measured with an SBS-KW-300SLIM scale. In addition, the PROMs (KOOS and BPI) will be collected during the research related control visits by the senior physiotherapist at previously defined time points. (Table 2)

Postoperative physiotherapy sessions will be provided by physiotherapists who will not otherwise be involved in the study. Physiotherapy control visits and rehabilitation guidance sessions with the treating physiotherapist will take place at 1, 4, 6, 8, 12 and 24 weeks. The physiotherapy protocol will last for 24 weeks. Protocols 1 and 2 are presented in the supplementary material.

Healing of the osteotomy site will be evaluated with plain radiograph examination at 6 and 12 weeks after the procedure. The radiographs will be evaluated by an experienced radiologist and the operating surgeons. Physician visits will be at 6 and 12 weeks. Knee scores will be assessed preoperatively and at 12 and 52 weeks postoperatively.

Complications

Complications will be categorised as follows:

- Infection
  - Superficial wound infection with or without positive bacterial culture. No need for revision surgery
  - Deep infection: Any infection with known bacterial source that requires revision surgery
- Implant failure leading to displacement of the osteotomy
- Non-union at 12 weeks control
- Persisting pain, CRPS or other reasons
- Need for revision surgery

Power analysis

We defined 10 degrees [[6](#_ENREF_6)] in knee ROM in the primary end-point as a clinically relevant difference. We estimated a standard population deviation of 20 degrees for knee ROM. Therefore, we need 63 patients per group to have a power of 80% with 5% type I error level. With this age group, the estimate of loss to follow-up rate will be set to 15% and will result in a total of 144 patients participating in the trial.

Statistical analysis

In the main analysis, the primary outcome will be analysed with linear regression using known prognostic and stratification variables as covariates in the analysis to reduce and handle the standard errors for the estimate of group difference. In addition to the stratification variables (age, C-D index), baseline ROM will be included in the main analysis as a covariate. A similar analysis will be performed for the secondary outcomes KOOS, muscle strength and BPII score. Each of the analyses will include respective baseline values as a third covariate. Unadjusted group difference will also be analysed using Welch t-test. Adjusted risk difference will be reported for complications and reoperations.

Data management plan

Electronic portfolios provided by the Redcap application will be used to collect patient research data. Patients will be pseudonymised: each patient will be assigned a unique trial identification number (TIN), which is then matched with the patient’s personal identification number. The identification of each patient will only be possible after retrieving the matching key, which will be stored in a locked locker in the research nurse’s office at Pihlajalinna Koskisairaala Hospital. Access to patient research data will not be given to parties outside the trial. Throughout the trial, the research data will only be handled with a TIN.

Data will be saved electronically by patients and research personnel to Tampere University Hospital’s secure research server – Redcap (via tablets and laptops), which has been security cleared by the hospital district. The research data saved to the server will contain only pseudonymous TINs with a set of numbers acquired from the questionnaires; that is, each question is answered with a number. This will ensure the anonymity of each individual patient, and that the identity of the patient will remain secret, even if server data are revealed to third parties.

Each researcher participating in the trial will gain access to the data at the end of the trial for further analyses. All variables in the data set will be described, and suitable metadata standards will be used, when available.

The copyright of the trial research data will be owned and created by the participating research parties. The data will be shared among all participating researchers who will receive access to the data after the trial is completed. Due to confidentiality and legal agreements, public data sharing will be restricted because we only have permission to hold the data in the specific research server, not to transfer data. Under certain circumstances, for example, when a new member joins the collaboration, we will grant access to the data.

Members of study group have access to the password-protected study data for analysis. All research team members are bound by professional secrecy with respect to identifiable patient information. The results of the study will be published in a form that does not identify those individuals who participated in the study.

Interim analysis

The external trial board will execute the interim analysis after half of the patients have been recruited. The analysis will focus on the number of adverse events (AEs) and the trial board, based on the results of the analysis, will give a recommendation as to whether the trial should continue. AEs and serious adverse events (SAEs) will be reported according to the recommendations given by the Consort Group.

AE is defined as follows:

- Superficial infection
- Persisting pain, CRPS or other reasons that can be cured on an ambulatory basis.

SAE is defined as follows:

- Deep infection: Any infection with known bacterial source that calls for re-operation
- Implant failure leading to displacement of the osteotomy
- Non-union at 12 weeks control visit

Trial schedule

Approval for the study will be sought in the spring of 2022. Patient recruitment will start in the summer of 2022. The study will document short-term recovery and the planned follow-up will be 1 year. The number of operations will be collected until the summer of 2023. Follow-up control visits will take place until the end of 2024.

After the 1-year follow-up, the results of the trial will be disseminated in a major peer-reviewed orthopaedic publication.

Ethics and dissemination

Ethical approval will be obtained from the local health authority. Patients recruited and those patients who declined to take part in the trial but joined the follow-up cohort will provide written informed consent. All data will be pseudonymised and the results will be published at the group level only. Individual patients will not be identifiable.

**Funding**

The trial does not have any external funding.

**Conflict of interest**

The research group members have no conflict of interests to declare.

References

1. Ambra LF, Phan A, Gomoll AH (2018) A New Technique for Distalization of the Tibial Tubercle That Allows Preservation of the Proximal Buttress. Orthop J Sports Med 6:2325967118798621

2. Biedert RM, Tscholl PM (2017) Patella Alta: A Comprehensive Review of Current Knowledge. Am J Orthop (Belle Mead NJ) 46:290-300

3. Caton J (1989) [Method of measuring the height of the patella]. Acta Orthop Belg 55:385-386

4. Caton J, Mironneau A, Walch G, Levigne C, Michel CR (1990) [Idiopathic high patella in adolescents. Apropos of 61 surgical cases]. Rev Chir Orthop Reparatrice Appar Mot 76:253-260

5. Insall J, Salvati E (1971) Patella position in the normal knee joint. Radiology 101:101-104

6. Insall JN, Dorr LD, Scott RD, Scott WN (1989) Rationale of the Knee Society clinical rating system. Clin Orthop Relat Res 13-14

7. Iwano T, Kurosawa H, Tokuyama H, Hoshikawa Y (1990) Roentgenographic and clinical findings of patellofemoral osteoarthrosis. With special reference to its relationship to femorotibial osteoarthrosis and etiologic factors. Clin Orthop Relat Res 190-197

8. Mansourvar M, Ismail MA, Raj RG, Kareem SA, Aik S, Gunalan R, et al. (2014) The applicability of Greulich and Pyle atlas to assess skeletal age for four ethnic groups. J Forensic Leg Med 22:26-29

9. Sherman SL, Erickson BJ, Cvetanovich GL, Chalmers PN, Farr J, 2nd, Bach BR, Jr., et al. (2014) Tibial Tuberosity Osteotomy: Indications, Techniques, and Outcomes. Am J Sports Med 42:2006-2017

10. Zwarenstein M, Treweek S, Gagnier JJ, Altman DG, Tunis S, Haynes B, et al. (2008) Improving the reporting of pragmatic trials: an extension of the CONSORT statement. BMJ 337:a2390
