# Supplementary material for: Effect of accelerated postoperative rehabilitation after tibial tubercle distalisation: A randomised controlled trial protocol
Source: PLoS One. 2024 Jul 11;19(7):e0304075. doi: 10.1371/journal.pone.0304075 (PMC11239065; doi:10.1371/journal.pone.0304075)
Supplement: Attachment — Submitted filename: Response to Reviewers 2.docx [file pone.0304075.s013.docx]

Reviewers' comments:

Reviewer's Responses to Questions

**Comments to the Author**

1. Does the manuscript provide a valid rationale for the proposed study, with clearly identified and justified research questions?

The research question outlined is expected to address a valid academic problem or topic and contribute to the base of knowledge in the field.

Reviewer #1: No

Reviewer #3: Yes

2. Is the protocol technically sound and planned in a manner that will lead to a meaningful outcome and allow testing the stated hypotheses?

The manuscript should describe the methods in sufficient detail to prevent undisclosed flexibility in the experimental procedure or analysis pipeline, including sufficient outcome-neutral conditions (e.g. necessary controls, absence of floor or ceiling effects) to test the proposed hypotheses and a statistical power analysis where applicable. As there may be aspects of the methodology and analysis which can only be refined once the work is undertaken, authors should outline potential assumptions and explicitly describe what aspects of the proposed analyses, if any, are exploratory.

Reviewer #1: No

Reviewer #3: Yes

3. Is the methodology feasible and described in sufficient detail to allow the work to be replicable?

Reviewer #1: No

Reviewer #3: Yes

4. Have the authors described where all data underlying the findings will be made available when the study is complete?

The [PLOS Data policy](https://journals.plos.org/plosone/s/materials-and-software-sharing) requires authors to make all data underlying the findings described in their manuscript fully available without restriction, with rare exception, at the time of publication. The data should be provided as part of the manuscript or its supporting information, or deposited to a public repository. For example, in addition to summary statistics, the data points behind means, medians and variance measures should be available. If there are restrictions on publicly sharing data—e.g. participant privacy or use of data from a third party—those must be specified.

Reviewer #1: No

Reviewer #3: Yes

5. Is the manuscript presented in an intelligible fashion and written in standard English?

*PLOS ONE* does not copyedit accepted manuscripts, so the language in submitted articles must be clear, correct, and unambiguous. Any typographical or grammatical errors should be corrected at revision, so please note any specific errors here.

Reviewer #1: Yes

Reviewer #3: Yes

6. Review Comments to the Author

Please use the space provided to explain your answers to the questions above and, if applicable, provide comments about issues authors must address before this protocol can be accepted for publication. You may also include additional comments for the author, including concerns about research or publication ethics.

You may also provide optional suggestions and comments to authors that they might find helpful in planning their study.

(Please upload your review as an attachment if it exceeds 20,000 characters)

Reviewer #1: The presented description is not aligned with the specific study, i.e. what method to prove what! Statsistical analysis dos not adress the point of analysis of the primary and secondary endpoint aligned with sample size justification. Analysis population is missing.

I recommended in my first review some aspects: The statistical analysis model has to be modified. The primary analysis is a stratified t-Test (Age) mit MI aggregation for missing. The sensitivity analysis may
include a mixed model for Repeated measures (MMRM, Mallinckrod et al (2013),
"Recent Developments in the Prevention and Treatment of Missing Data")) using the
longitudinal observations. The latter should be used for the secondary outcomes as
well.

*Thank you for the remark. However, we humbly disagree with the reviewer. Our study involves a repeated design, and therefore, the statistical approach should consider this appropriately. We refer to following;*

*Studies have shown (eg.* [*https://www.tandfonline.com/doi/full/10.1080/10543401003777995*](https://www.tandfonline.com/doi/full/10.1080/10543401003777995)*) that MMRM performed better than MI on longitudinal data.*

*Additionally, the EMA guideline is also agnostic to the choice of the model (*[*https://www.ema.europa.eu/en/documents/scientific-guideline/guideline-missing-data-confirmatory-clinical-trials_en.pdf*](https://www.ema.europa.eu/en/documents/scientific-guideline/guideline-missing-data-confirmatory-clinical-trials_en.pdf)*).*

*Finally, MI is also not needed MMRM (e.g.* [*https://www.jclinepi.com/article/S0895-4356(13)00123-6/*](https://www.jclinepi.com/article/S0895-4356(13)00123-6/)*).*

*Due to the previous arguments, we would leave the analysis as it is. However, we leave it to the Editor to decide and will change it accordingly, if needed.*

Reviewer #3: congratulation
your manuscript is acceptable............................................................................

7. PLOS authors have the option to publish the peer review history of their article ([what does this mean?](https://journals.plos.org/plosone/s/editorial-and-peer-review-process#loc-peer-review-history)). If published, this will include your full peer review and any attached files.


If you choose “no”, your identity will remain anonymous but your review may still be made public.


**Do you want your identity to be public for this peer review?** For information about this choice, including consent withdrawal, please see our [Privacy Policy](https://www.plos.org/privacy-policy).

Reviewer #1: No

Reviewer #3: **Yes:** Soheil Mansour Sohani
